# Absence of enterotypes in the human gut microbiomes reanalyzed with non-linear dimensionality reduction methods

**DOI:** 10.7717/peerj.15838

**Published:** 2023-09-08

**Authors:** Ivan Bulygin, Vladislav Shatov, Anton Rykachevskiy, Arsenii Raiko, Alexander Bernstein, Evgeny Burnaev, Mikhail S. Gelfand

**Affiliations:** 1 Skolkovo Institute of Science and Technology, Moscow, Russia; 2 Moscow State University, Moscow, Russia; 3 Artificial Intelligence Research Institute (AIRI), Moscow, Russia; 4 Institute for Information Transmission Problems, Moscow, Russia

**Keywords:** Human gut microbiome, Dimensionality reduction, Clustering, Enterotypes

## Abstract

Enterotypes of the human gut microbiome have been proposed to be a powerful prognostic tool to evaluate the correlation between lifestyle, nutrition, and disease. However, the number of enterotypes suggested in the literature ranged from two to four. The growth of available metagenome data and the use of exact, non-linear methods of data analysis challenges the very concept of clusters in the multidimensional space of bacterial microbiomes. Using several published human gut microbiome datasets of variable 16S rRNA regions, we demonstrate the presence of a lower-dimensional structure in the microbiome space, with high-dimensional data concentrated near a low-dimensional non-linear submanifold, but the absence of distinct and stable clusters that could represent enterotypes. This observation is robust with regard to diverse combinations of dimensionality reduction techniques and clustering algorithms.

## Introduction

The human gut is populated by a diverse community of microorganisms. The microbiome of an individual gut settles in several years after birth and, by rough estimates, contains more than a thousand genera of bacteria (Gilbert et al., 2018). Gut microbiota forms a dynamic ecosystem whose composition tends to be constant during the life of an individual but varies between individuals and may significantly depend on external and internal factors. The initial sequencing of the gut biota revealed that its composition tends to form discrete groups (enterotypes) consisting predominantly of taxa *Bacteroides*, *Prevotella*, and *Ruminococcus* (Arumugam et al., 2011). Enterotypes have been reported in Arumugam et al. (2011) as “densely populated areas in a multidimensional space of community composition” which “are not as sharply delimited as, for example, human blood groups”.

**Figure 1:**
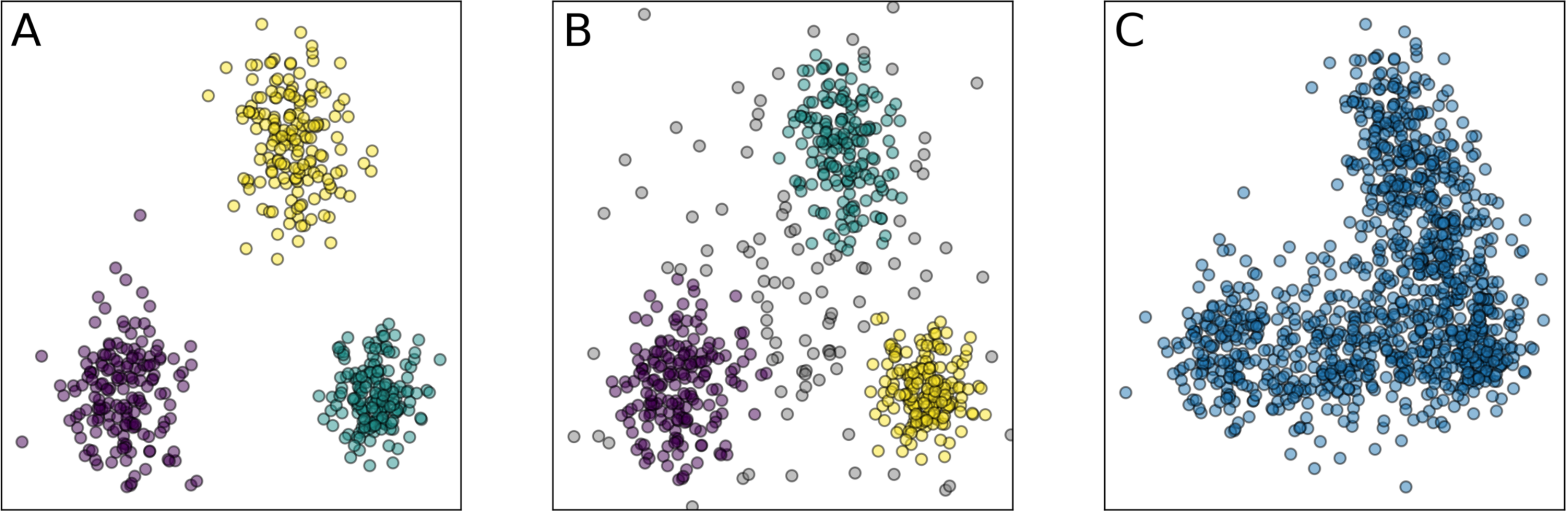
Examples of the clustering partitions. (A) Clusters as well-separated sets of points; (B) clusters as regions of points with a higher density than the background; (C) absence of clusters, but presence of the low-dimensional structure. Similar distinction also applies for non-convex clusters. Colors indicate different clusters.

In Costea et al. (2017) this concept was revisited, suggesting a more careful definition reflecting non-discrete structure and non-uniform density of the microbial composition. Still, many recent papers consider enterotypes as discrete clusters in the relative taxonomic abundance, and here we attempt to follow this approach, but in a more rigorous way. From a geometric point of view, clusters are defined as dense areas separated by sparse regions. Clustering is a process of assigning a finite set of objects to separate groups and identifying the natural structure of the data when the relationship between objects is represented as a metric, *e.g.*, the Euclidean distance (Maronna, Charu & Chandan, 2015). Enterotypes may be defined either as well-separated clusters without points between them or as regions with a higher density of data points, indicating preferential clustering, as shown in Figs. 1A and 1B, respectively. We address both cases using diverse methods and metrics, sensitive for either the first or both types of clusters.

A major current challenge is to determine the existence of enterotypes *via* “a thorough quantitative investigation of established clustering methods and tests for microbiome data” (Knights et al., 2014). Published studies rely on similar approaches yet differ in the exact number of enterotypes ranging from two (Li et al., 2018; Yin et al., 2017; Chen et al., 2017; Nakayama et al., 2017; Kang et al., 2016; Nakayama et al., 2015; Wang et al., 2014; Ou et al., 2013; Wu et al., 2011) to three (Wu et al., 2017; De Moraes et al., 2017; Vieira-Silva et al., 2016; Emoto et al., 2016; Robles-Alonso & Guarner, 2013) and four (Gotoda, 2015). In most papers the presence of enterotypes in the microbiome data is determined by clustering of the data into *K* groups, with *K* selected by optimization of some metric of the clustering partition quality. The resulting segregation is then visualized in the projection to the first two or three principal components from the principal components analysis (PCA). This approach is general and may involve various intermediate steps, different clustering algorithms, and a variety of metrics; however, it has certain flaws. For example, the PAM method used in many studies may yield erroneous results for density-based clusters. Also, widely used in the studies partition quality metrics such as the Calinski–Harabasz Index  (Calinski & Harabasz, 1974) and the Silhouette score (Rousseeuw, 1987) are naturally higher for convex clusters and may fail to detect density-based partition since they rely on the estimation of inter-cluster variance and cluster centers.

Another common problem is the small size of datasets in comparison to their dimensionality. The direct application of standard clustering methods for small and high-dimensional datasets, performed in most of the works, may lead to unreliable results due to the curse of dimensionality. Clustering implies the notion of dissimilarity between data samples. When the data dimensionality increases, the concepts of proximity, distance, or nearest neighbor become less qualitatively meaningful, especially for the commonly used Euclidean or Manhattan distances (Aggarwal, Hinneburg & Keim, 2001). For example, the distance to the nearest data point approaches the distance to the farthest data point (Beyer et al., 1999). The lack of data further amplifies this problem since the data point cloud in a high-dimensional space becomes sparse, yielding unreliable estimates of the probability density due to the non-asymptotic lower bound for the regression error (Kohler, Krzyzak & Walk, 2009; Ibragimov & Has’minskii, 1981). One straightforward way to overcome it is to reduce the data dimensionality by PCA. However, this would allow one only to find an affine subspace containing most of the data variance. It may not be sufficient to effectively decrease the data dimensionality without significant loss of information when the data lies near a non-linear low-dimensional manifold.

Inconsistency in the number of enterotypes found in different works and the aggravating factors described above undermine the very notion of enterotypes. Several studies demonstrated the possibility of a gradient distribution and the absence of well-defined clusters (Jeffery et al., 2012; Yatsunenko et al., 2012; Claesson et al., 2012; Cheng & Ning, 2019; Costea et al., 2017). Different structures of enterotypes between males and females were claimed by Oki et al. (2016). In several works, the very concept of enterotypes was described as inconsistent and uncertain (Knights et al., 2014; Jeffery et al., 2012). Factors such as the variation in the microbial load between samples (Vandeputte et al., 2017), robustness of enterotypes clusters (Huss, 2014), and microbiome variation during short periods of time (Knights et al., 2014) were considered limiting to the use of the enterotype concept. However, enterotyping of the human gut has been applied in clinical research. Published studies claimed correlations between enterotypes and diet (Chen et al., 2017; Nakayama et al., 2017; Kang et al., 2016; Nakayama et al., 2015; Wu et al., 2011; Klimenko et al., 2018; Shankar et al., 2017; Liang et al., 2017; Xia et al., 2013), inflammatory gut diseases (Vieira-Silva et al., 2019; Castaño Rodríguez et al., 2018; Moser, Fournier & Peter, 2017; Dlugosz et al., 2015; Chiu et al., 2014; Huang et al., 2014; De Wouters, Doré & Lepage, 2012), mental health (Lee et al., 2020), acne (Deng et al., 2018), stool composition (Tigchelaar et al., 2015; Vandeputte et al., 2015), colorectal cancer (Thomas et al., 2016), circulatory diseases  (Emoto et al., 2016; Jie et al., 2017; Franco-de Moraes et al., 2017), psoriasis (Codoñer et al., 2018), and infections such as AIDS (Noguera-Julian et al., 2016) and influenza (Qin et al., 2015; Shortt et al., 2018). The idea that information about the enterotype of an individual may be a helpful biomarker not only to correct gut diseases but also to aid other medical interventions (Gilbert et al., 2018) relies on the assumption that enterotypes are discontinuous clusters that are stable in time at least on the short scale; this has been challenged recently  (Cheng & Ning, 2019).

Here, we look for balanced, stable, and distinct clusters in large stool microbiome datasets. Balance implies that each cluster should contain a sufficient number of points to be considered as a potential enterotype. Stability means that clusters should not depend on data bootstrapping, and transformations that preserve the general form of the data point cloud. Meaningful clusters should not disappear if the dataset is changed in a non-essential way. Partition of the data into distinctive clusters should correspond to high values of an appropriate clustering validity index that is applicable both for convex and density-based clusters. In other words, there should be separating gaps seen as space regions with a lower concentration of points, whereas clusters correspond to areas of highest concentration. All these requirements are addressed by appropriate metrics and methods, described in ‘Materials & Methods’. To ensure accurate analysis of the high-dimensional data, we introduce two new intermediate steps into the common pipeline for the microbiome clustering analysis: estimation of the intrinsic dimension and manifold learning. These steps allowed us to significantly reduce data dimensionality while preserving most of the information. Using such low-dimensional representation of the data, we demonstrate the absence of stable and distinct clusters in several large datasets of 16S rRNA-genotyped stool samples. This absence of natural clusters is seen in the example in Fig. 1C.

## Materials & Methods

As the main source of the human gut microbiome data, we used the 16S rRNA genotype data from the NIH Human Microbiome Project (HMP) (The Human Microbiome Project Consortium, 2012a; The Human Microbiome Project Consortium, 2012b) and American Gut Project (AGP) (McDonald et al., 2018). These largest available datasets provide a sufficient number of data points for correct estimation of the clustering partition and constructing a manifold (Psutka & Psutka, 2015). Both datasets were collected in the United States. No patterns driven by geography or lifestyle were explicitly taken into account in our framework. We did not use longitudinal sampled microbiota datasets, as we were not concentrating on the dynamics of enterotypes, but rather on their existence. Similarly, we did not use shotgun sequencing data (Pasolli et al., 2017), as its characterized taxonomy composition is limited to sequenced genomes. We used 4,587 HMP samples from stool and rectum body sites downloaded from the Human Microbiome Project (https://portal.hmpdacc.org) and 9,511 samples from AGP downloaded from figshare (https://figshare.com/articles/dataset/American_Gut_Project_fecal_sOTU_relative_abundance_table/6137198) as abundance matrices. For comparison with the original research (Arumugam et al., 2011), we analyzed Sanger (Gill et al., 2006), Illumina (Qin et al., 2010), and Pyroseq (Turnbaugh et al., 2008) datasets from (http://www.bork.embl.de/Docu/Arumugam_et_al_2011/). The results are presented in Text S3, Table S2, Figs. S2–S5. All datasets were normalized by dividing Operational Taxonomic Units (OTUs) values by the total sum of abundances for a given data sample. An OTU found in less than 1% of the samples or with a standard deviation less than 0.001 were removed to ease the preprocessing step. To account for outliers with microbiome dominated by single or few species, *e.g.*, in patients with extreme gut microbiota we repeated the analysis, for the HMP and AGP datasets with removed OTUs accounting for >70% abundance. The results were consistent, see Text S5, Tables S3–S4, Figs. S8–S15.

As the first step of dimensionality reduction after preprocessing, we use PCA to identify a medium-dimensional linear subspace retaining almost all data cloud variation. The dataset projected on this subspace does not significantly differ from the original dataset, while removing dimensions with low variance acts as a filter that provides a more robust clustering (Ben-Hur & Guyon, 2003). We preserve the variances after projection, since removing them may hinder the subsequent clustering process and lead to erroneous results. Instead of limiting the dimensionality reduction process solely to PCA, as in previous studies, we then determine the intrinsic dimension of the projected data *via* the maximum likelihood estimation (MLE) (Levina & Bickel, 2004). This step allows for capturing a minimal but sufficient number of coordinates representing the most important features of the dataset. Following the manifold hypothesis (Fefferman, Mitter & Narayanan, 2016), we suppose that a microbial data cloud lies near some lower-dimensional manifold embedded in the high-dimensional abundance space. The goal of non-linear manifold learning is to obtain a low-dimensional representation of the data, supposedly lying on such a manifold, while preserving most of the information. This information may be expressed as similarities or dissimilarities between data points, *e.g.*, as a matrix of pairwise distances. At that non-linear projections per se are not interesting, since any data cloud could perfectly lie on a one-dimensional submanifold.

While this one-dimensional submanifold yields a perfect alignment in terms of minimization of the distance between the original data point and its projection, it does not preserve information in terms of pairwise distances. Therefore, it is important to assess the quality of embeddings provided by manifold learning algorithms. Proper embedding should preserve local and global structure, *e.g.*, points that are close in the original space should remain close in the embedding space. Given the intrinsic dimension, we further reduce data dimensionality using several manifold learning algorithms, namely: Isomap (Tenenbaum, De Silva & Langford, 2000), locally linear embedding (LLE) (Zhang & Wang, 2007), denoising autoencoder (AE) (Goodfellow, Bengio & Courville, 2016), spectral embedding (SE) (Shi & Malik, 2000), t-distributed Stochastic Neighbor Embedding (t-SNE) (Van Der Maaten & Hinton, 2008), and uniform manifold approximation and projection (UMAP) (McInnes et al., 2018). A detailed description of these algorithms and their pros and cons is beyond the scope of this article. These methods are conceptually different and susceptible mostly for quantitative comparison, rather than qualitative. A short description is provided in Table S5. For each manifold learning algorithm, dataset, and taxonomic level, we obtain a low-dimensional embedding. To find the near-optimal hyperparameters of the manifold learning algorithm for a specific dataset and taxonomic level, we iterate over various combinations of hyperparameters. For each combination, we assess how well an embedding produced by an algorithm with this combination of parameters, represents the original data.

We selected a computationally feasible hyperparameters range, with reasonable values, according to our expectation of the number of clusters and common machine learning practice. To ensure reproducibility of the results, we restricted the number of hyperparameter combinations from eight to 40, depending on the algorithm. For details on the training procedures and hyperparameters choice, refer to the software link in the ‘Data Availability’ section.

To compare the dimensionality reduction algorithms with regard to the loss of information, we construct an inverse mapping from the obtained low-dimensional manifold back to the original space using k-nearest neighbors regression (Fix & Hodges, 1989) with five nearest neighbors and distance weighting. Then, we estimate the reconstruction error using the Leave-One-Out procedure (Ruppert, 2004). We report the resulting error as median of the absolute error (MAE) across all reconstructed points. This technique assesses how well points coordinates in the original space can be reconstructed given their neighbors from the embedding space. Inverse mapping from the embedding to the original space is usually performed by a supervised learning algorithm that minimizes the reconstruction error. Selecting an algorithm, its hyperparameters, and different ways to train it would bring ambiguity into our method. Moreover, the MAE alone is an intractable metric that does not show what aspect of the data cloud has been misrepresented. Therefore, we apply two additional criteria of quality of dimensionality reduction (Lee & Verleysen, 2010). These criteria, *Q*_*loc*_ and *Q*_*glob*_, represent the preservation of the “local” and the “global” structure and are described in Text S1. It should be noted that *Q*_*glob*_ is a more important metric for the studied problem since local distortion of the data should not significantly impact the clustering partition that may be implicitly present in the data. Given hyperparameters that deliver the lowest MAE, we iteratively discard up to 10% of initial data points with the highest reconstruction MAE, which serves as denoising for a more stable clustering in the embedding space. It does not affect the clustering partition results, since these data points are outliers as determined by the Local Outlier Factor algorithm (Breunig et al., 2000). The latter allows for detecting outliers by deviation of their local density with respect to their neighbors, from which it follows that they are not related to any cluster as the latter are densely populated areas in a multidimensional space.

For each low-dimensional embedding, we apply several clustering methods-Spectral Clustering (Shi & Malik, 2000; Von Luxburg, 2007), PAM, and Hierarchical Density-Based Spatial Clustering of Applications with Noise (HDBSCAN)  (McInnes & Healy, 2017). A detailed description of these algorithms and their pros and cons is beyond the scope of this article, while a short description is provided in Table S6. HDBSCAN and spectral clustering are useful when the structure of the clusters is arbitrarily shaped and non-convex. We use PAM as a baseline and for comparison with related works. For each clustering method, we iterate over a set of hyperparameter combinations to find a partition that yields the best clustering validity metrics. As a result, we obtain more robust results not biased by the peculiarities of the algorithms and the choice of hyperparameters.

Clustering metrics based on the ratio of within-cluster compactness to between-cluster separation, like the Calinski–Harabasz index (Calinski & Harabasz, 1974), the Silhouette score (Rousseeuw, 1987), and the Davies–Bouldin index (Davies & Bouldin, 1979) cannot handle arbitrarily shaped clusters and noise in the form of low-density points scattered around dense clusters areas. Thus, as the main metric for clustering validity, we consider Density-Based Clustering Validation (DBCV) (Moulavi et al., 2014), which accounts for both density and shape properties of clusters, tolerates noise, and is appropriate for detecting density-based clusters. We assess the clustering partition stability using prediction strength (Tibshirani & Walther, 2005) initially proposed for estimating the number of clusters, which tells us how well the decision boundaries of the clustering partition, calculated on a data subset, generalize the data distribution. In addition, to be considered as a stable enterotype, a cluster should contain a sufficiently large number of data points. Hence, we do not consider spurious clusters that contain less than 5% of data assuming that such clusters are outliers or artifacts of dimensionality reduction algorithms. Indeed, they are small, depend on manifold learning algorithms, and are separated from the main data point cloud. Nevertheless, they significantly impact the clustering quality metrics. To detect such imbalanced partitions, we use Shannon Entropy (Shannon, 1948) of the probability distribution of data points to be in a certain cluster. All these metrics are described in detail in Text S1.

**Figure 2:**
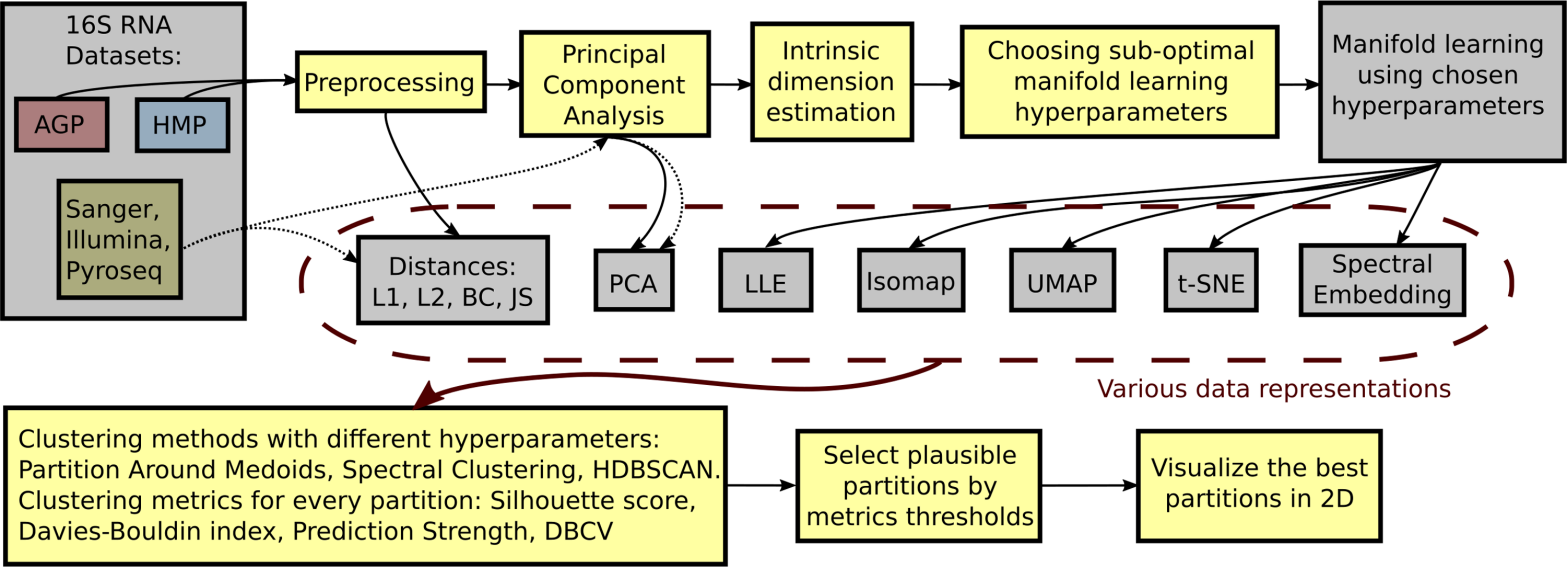
Schematic depiction of our framework for clustering high-dimensional data. This procedure is applied to each dataset (AGP, HMP) at every taxonomy level (O, Order; F, Family; G, Genus). The data is preprocessed by removing OTUs that are found in less than 1% of the samples or have a standard deviation less than 0.001. For the preprocessed data, pairwise distances are calculated: Manhattan (L1), Euclidean (L2), Bray-Curtis (BC), and Jensen–Shannon (JS). PCA representation is obtained by projecting the data onto principal components explaining 99% variance. After such projection, the intrinsic dimension of the data is estimated. The intrinsic dimension for every dataset at different taxonomy levels is given, and suboptimal hyperparameters for every manifold learning algorithm are found by minimizing the reconstruction median absolute error over different hyperparameter combinations. Then, the collection of data representations is extended by adding the results of nonlinear dimensionality reduction methods obtained using the found suboptimal hyperparameters. For every data representation in the collection, various clustering algorithms with different hyperparameters are applied. Partition Around Medoid (PAM), spectral clustering, and HDBSCAN. Then, for every found partition, clustering metrics are assessed. As a result, only partitions that pass certain metrics thresholds are considered plausible.

To identify the optimal clustering, we compare DBCV, prediction strength, and entropy for each partition respective to different clustering hyperparameters. A balanced clustering partition that corresponds to separation of the data cloud into distinct and stable clusters should produce a salient local maximum of the DBCV score, Entropy value, and Prediction Strength. Also, following previous studies, for each clustering partition, we calculate the Davies–Bouldin Index and the Silhouette score. Lower Davies–Bouldin Index and higher Silhouette score correspond to better partitions, where clusters are better in terms of compactness and separation. We summarize all steps described above in a single framework schematically shown in Fig. 2.

To demonstrate the continuous nature of the stool microbial data distribution, we construct 2D and 3D coordinate projections of the data using t-distributed stochastic neighbor embedding (t-SNE) (Van Der Maaten & Hinton, 2008) and UMAP algorithms (McInnes et al., 2018). For most dimensionality reduction methods, validity indices, and metrics, implementations from the ‘scikit-learn‘ library (Pedregosa et al., 2011) were used. For the HDBSCAN clustering method and the DBCV metric the ‘hdbscan‘ package (McInnes & Healy, 2017) from the ‘scikit-learn-contrib‘ was used. For the UMAP algorithm we applied the implementation from the ‘umap-learn‘ library (McInnes et al., 2018).

## Results

### Data preprocessing

**Figure 3:**
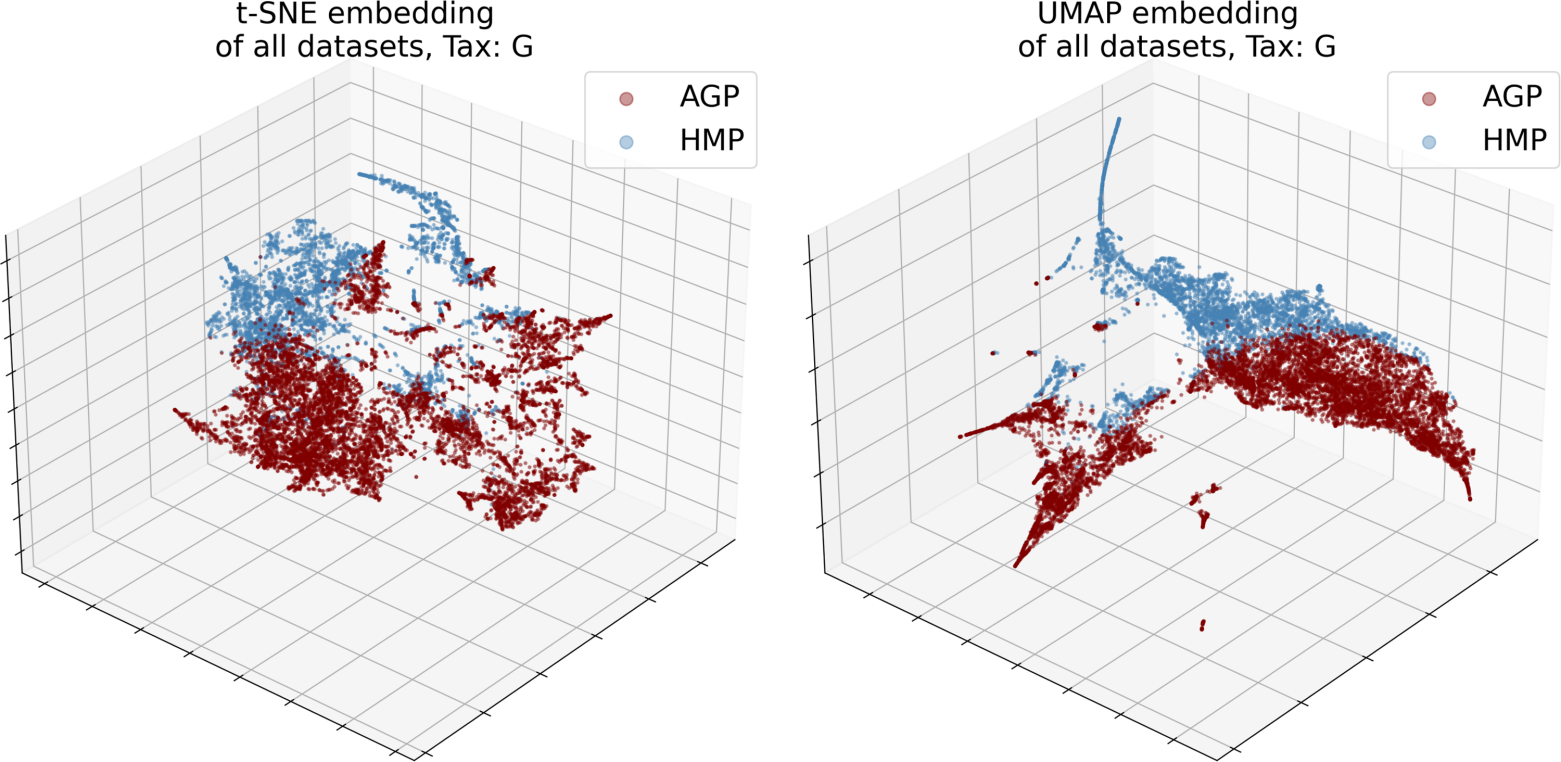
3D t-SNE and UMAP visualizations of joined datasets demonstrate batch-effect at Genus taxonomy level (tax). Red—AGP, green—HMP dataset.

The numbers of objects for both datasets and their dimensionality *d* in the relative taxon abundance space, before and after preprocessing, are presented in Table 1. The datasets were analyzed at the Order, Family, and Genus taxonomic levels (denoted O, F, and G, respectively). The data distribution is inherently sparse due to the insufficient number of samples and noisy due to the presence of possible outliers. Noise may correspond to specific patients with exotic microbial communities or be caused by sample collection or data processing artifacts. Moreover, the procedures may vary between laboratories, leading to considerable batch effects; for instance, dataset-specific preprocessing has been applied to correct for microbial blooms in the AGP dataset (Amir et al., 2017). Therefore, we cannot merge several individual datasets into one. Indeed, visualization using t-SNE (Van Der Maaten & Hinton, 2008) and UMAP (McInnes et al., 2018) dimensionality reduction algorithms illustrates that point in Fig. 3. Hence, to avoid the batch effect, we considered these datasets separately.

**Table 1:** Size and dimensionality d of the AGP and HMP datasets in Order, Family, and Genus taxonomic levels. Initial (init.) dimensionality corresponds to raw data and processed (proc.) corresponds to data, after removing OTU found in less than 1% of the samples or with a standard deviation less than 0.001.

**Dataset**	**Size**	**Order** *d*		**Family** *d*		**Genus** *d*	
		**init.**	**proc.**	**init.**	**proc.**	**init.**	**proc.**
**AGP**	9511	168	39	258	69	535	108
**HMP**	4587	179	39	267	70	574	97

### Principal Component Analysis (PCA)

**Figure 4:**
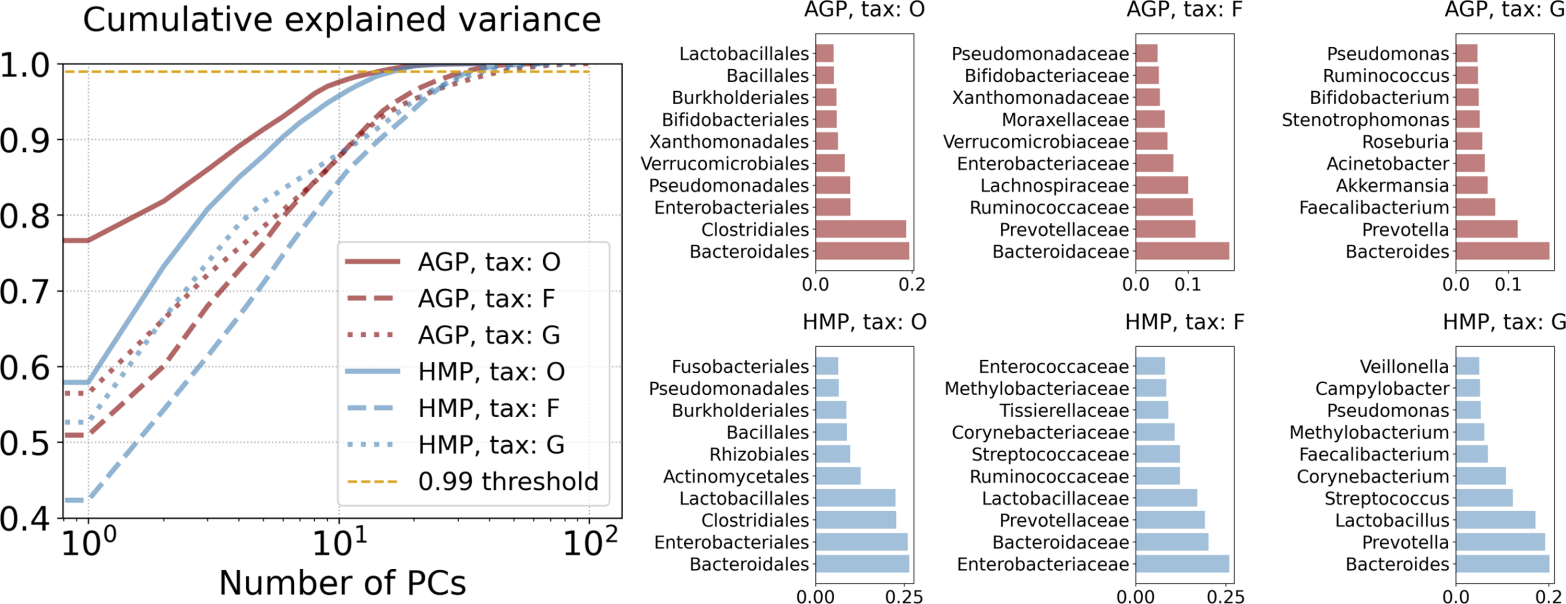
Principal components analysis. Cumulative explained variance and PCA loadings, representing contribution of the original taxonomy coordinates to the principal components.

We obtained significant dimensionality reduction with minuscule information loss by using projection on relatively many (16 through 47, dependent on the taxonomy level) principal components. Dimensionalities *d*_*PCA*_ of the PCA projections are reported in Table 2 and defined as the number of first principal components that meet the selected threshold of 99% of the cumulative explained variance. Contribution of original taxonomic coordinates to the principal components, also known as the PCA loadings, can be calculated as the Euclidean norm of the corresponding principal vectors coordinates multiplied by the square root of the associated eigenvalue. The cumulative explained variance and PCA loadings are presented in Fig. 4. Evidently, for both datasets at the Genus level *Bacteroides* and *Prevotella* contribute the most to the variance and the resulting PCA components. At the Family level it is again *Bacteroidaceae* and *Prevotellaceae* for both datasets, but with *Ruminococcaceae* for AGP and *Enterobacteriaceae* for HMP as additional strong drivers of the variance. At the Order level, the variance is dominated by *Bacteroidales, Enterobacteriales*, and *Clostridiales* for both datasets. To assess the information loss during projection, we calculated the error of reconstruction from the projected data to the original one. The Median Absolute Error (MAE) and *Q*_*loc*_ and *Q*_*glob*_ metrics (for details see Text S1) are presented in Table 3. Reconstruction of the original data from the data projected on principal components was obtained using an inverse linear transformation.

**Table 2:** Dimensionalities *d*_*PCA*_ and *d*_*MLE*_ of two datasets (AGP and HMP) in three taxonomy levels (O, Order; F, Family; G, Genus).

**Dataset**	**Tax O**		**Tax F**		**Tax G**	
	***d***_*PCA*_	***d***_*MLE*_	***d***_*PCA*_	***d***_*MLE*_	***d***_*PCA*_	***d***_*MLE*_
**AGP**	16	6	34	8	47	8
**HMP**	18	5	35	6	40	6

**Notes.**

*d*_*PCA*_ number of the first principal components explaining 99% variance *d*_*MLE*_ estimated intrinsic dimension.

**Table 3:** PCA dimensionality reduction metrics. Median absolute error (MAE) of the linear inverse transformation from the data projected on the principal components to the original space of taxon abundances. Q_loc_ and Q_glob_ metrics denote preservation of the local and global data structure (see the text for details). Notation as in Table 2.

**Dataset**	**Tax**	**MAE**	***Q***_*loc*_	***Q***_*glob*_
**AGP**	**O**	0.061	0.90	0.99
	**F**	0.041	0.96	0.99
	**G**	0.050	0.95	0.99
**HMP**	**O**	0.036	0.91	0.99
	**F**	0.044	0.92	0.98
	**G**	0.058	0.92	0.99

### Estimation of the intrinsic dimension

We calculated the intrinsic dimensions *d*_*MLE*_ for each dataset at each taxonomic level by applying the Maximum Likelihood Estimation principle to the distances between close neighbors (Levina & Bickel, 2004). As a dimension estimation we have selected the median of the intrinsic dimension distribution across the neighborhood cardinality, which varies from 5 to 100. The distribution is estimated using bootstrapping technique for 50 trials. The resulting intrinsic dimensions and the dimensionality after projection on principal components are presented in Table 2.

### Manifold learning

**Figure 5:**
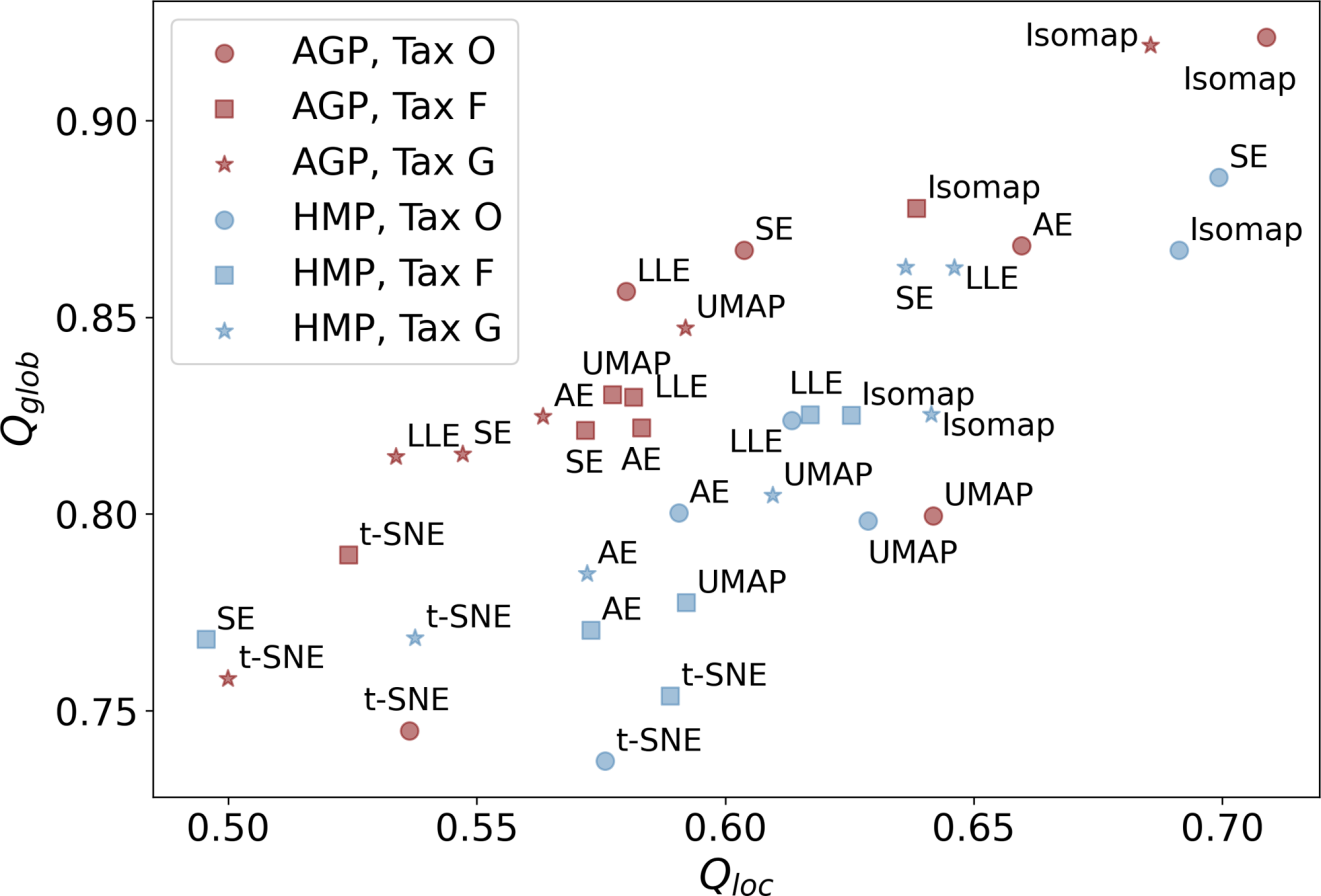
Metrics of the data structure preservation for different dimensionality reduction methods, taxonomy levels and datasets. Horizontal axis-Q_loc_ metric (local information preservation), vertical axis-Q_glob_ metric (global information preservation) of the non-linear dimensionality reduction methods. Datasets (AGP and HMP) and taxonomy levels (O, Order; F, Family; G, Genus) are shown in the inset. SE, spectral embedding, LLE, locally linear embedding, AE, autoencoder.

Subsequent non-linear dimensionality reduction from *d*_*PCA*_to *d*_*MLE*_, is performed by different manifold learning algorithms described in the Materials & Methods section and Table S5. In Fig. 5, we present the *Q*_*loc*_, and *Q*_*glob*_ metrics that represent the preservation of the local and global data structure after dimensionality reduction. They were evaluated for all manifold learning methods with near-optimal hyperparameters, applied to all datasets at different taxonomic levels. Near-optimal hyperparameters were found using manifold learning algorithms with different combinations of potential hyperparameters and selecting the ones with the lowest reconstruction MAE. The corresponding MAE values of the original data reconstruction from a nonlinear embedding are listed in Table 4. It shows that higher taxonomy levels yield more intricate data representation with higher MAE and lower *Q*_*loc*_, *Q*_*glob*_. This is due to data becoming sparser and more dissipated in high-dimensional space, which hinders data representation by fitting a non-linear manifold.

**Table 4:** Median Absolute Error (MAE) of the data reconstruction from different manifold learning embeddings. MAE is assessed using Leave-One-Out procedure. The reconstruction is done by the independent k-nearest neighbors regression of the coordinates in the original space of relative taxon abundances from the non-linear embedding. Notation as in Table 2.

**Dataset**	**Method**	**Tax O**	**Tax F**	**Tax G**
**AGP**	AutoEncoder	0.06	0.19	0.22
	t-SNE	0.05	0.20	0.22
	UMAP	0.06	0.22	0.24
	Isomap	0.06	0.22	0.25
	LLE	0.06	0.21	0.24
	Spectral	0.06	0.21	0.24
**HMP**	AutoEncoder	0.09	0.24	0.22
	t-SNE	0.09	0.24	0.22
	UMAP	0.11	0.27	0.25
	Isomap	0.11	0.29	0.27
	LLE	0.12	0.29	0.26
	Spectral	0.13	0.34	0.29

### Clustering

**Figure 6:**
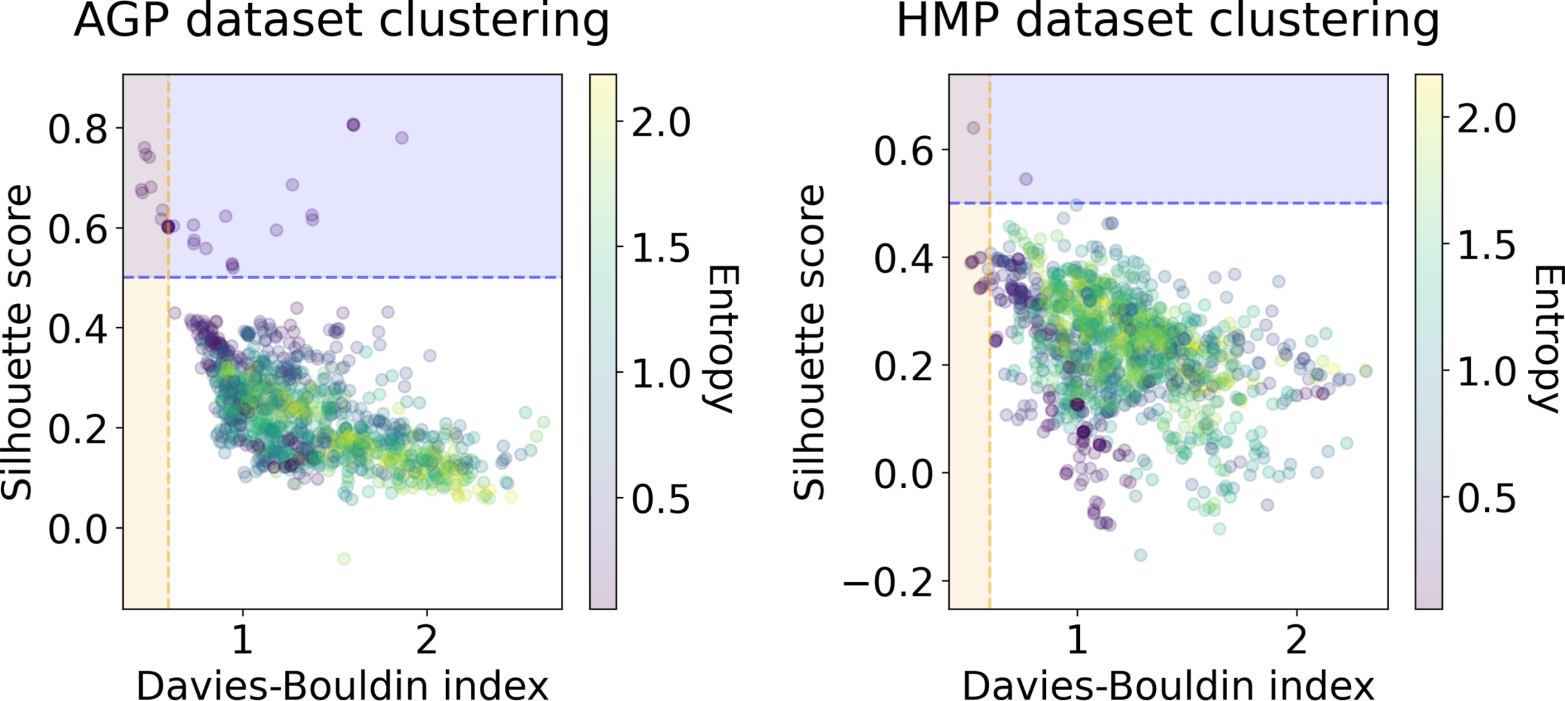
Silhouette score and Davies–Bouldin Index of the clustering results for the AGP and HMP datasets. All three taxonomy levels (O, Order; F, Family, G, Genus) are displayed. The point color represents the entropy of the respective partition.

**Figure 7:**
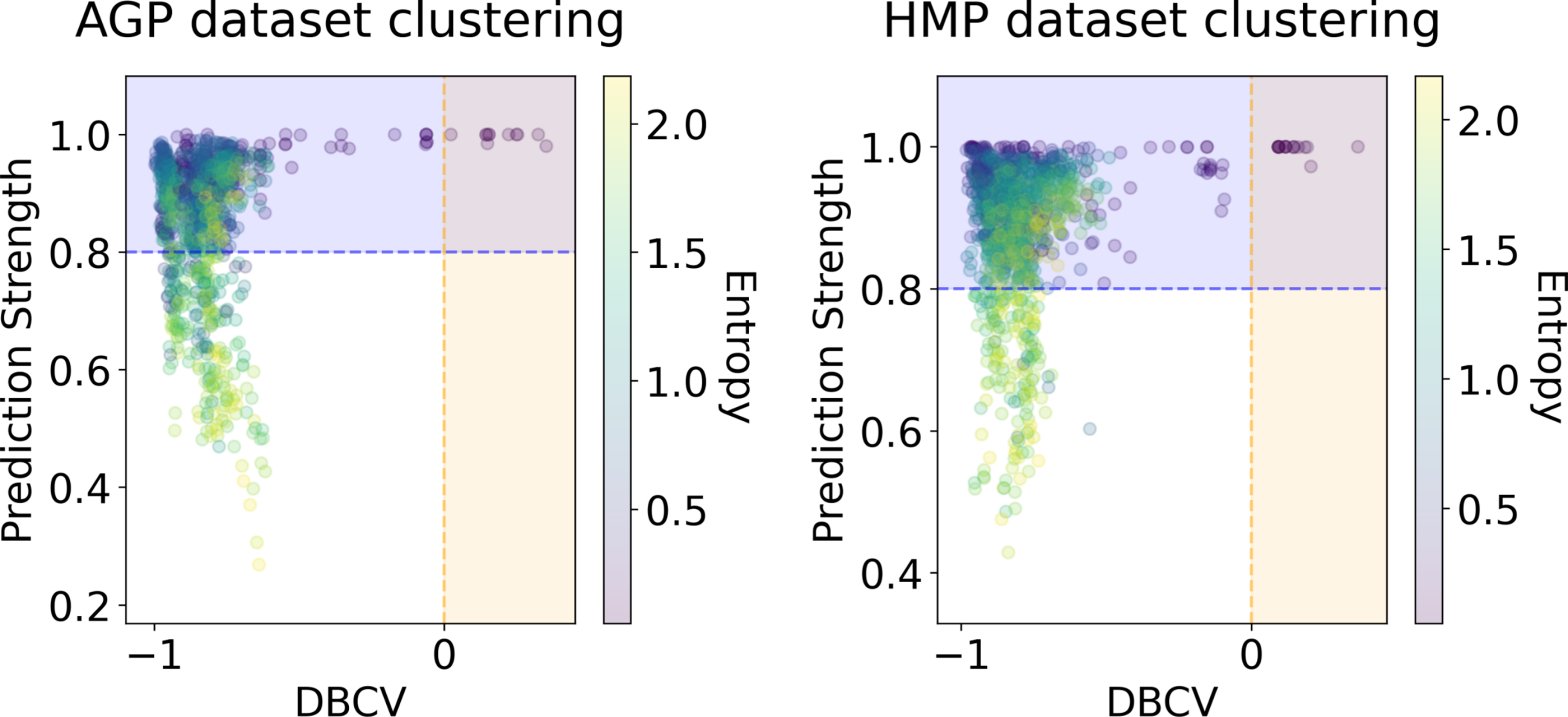
DBCV index and the prediction strength of the clustering results for the AGP and HMP datasets. All three taxonomy levels (O, Order; F, Family; G, Genus) are displayed. The point color represents the entropy of the respective partition.

We applied several clustering methods using the Euclidean metric - Spectral Clustering (Von Luxburg, 2007), PAM and HDBSCAN (McInnes & Healy, 2017)-for each embedding provided by the dimensionality reduction algorithms. Following related works on the identification of enterotypes, we also applied clustering to the original data in high-dimensional space of taxonomic abundances with a variety of distance metrics: Jensen–Shannon, Manhattan, Euclidean, and Bray-Curtis as in Koren et al. (2013). It should be noted that we used only the Euclidean and Manhattan metric in the manifold learning algorithms since distribution-based distances such as Jensen–Shannon are not applicable after the PCA projection. Originally, *Bacteroides, Prevotella*, and *Ruminococcus* have been considered as the main drivers of microbial variation that contribute most to the enterotypes (Arumugam et al., 2011). To ensure that there is no evident clustering structure within these genera, we visualize the three-dimensional distribution of the normalized abundances of these OTUs in Fig. S6 (top). We observe a continuous distribution with no apparent natural clusters. We support this observation, by adding this three-dimensional projection of the datasets into the pool of all representations to which we apply clustering methods. Such representations are comprised by manifold-learning embeddings, original data in different metric spaces, and data projected on principal components. The resulting metrics of all clustering results in different data representations are shown in Figs. 6 and 7. To distinguish between the presence and absence of clusters in the data we consider the following thresholds. For the Prediction Strength, we consider a score of 0.8 for moderate support as suggested in Tibshirani & Walther (2005) and Koren et al. (2013). We consider all positive values of the DBCV metric. For the Silhouette score, we consider a score of 0.5 for moderate clustering as suggested in Wu et al. (2011); Koren et al. (2013); Gentle, Kaufman & Rousseuw (1991); Arbelaitz et al. (2013). As a threshold value for the Davies–Bouldin index, we used 0.6 for moderate clustering  (Davies & Bouldin, 1979). Under the assumption that our data capture all microbiome variations that may be possibly related to enterotypes, we do not consider small clusters that contain less than 5% of the data as natural clusters related to enterotypes.

To validate the ability of our methods to provide accurate clustering of high dimensional data, we also provide results on simple synthetic datasets (for details see Text S2). Since the clustering results directly depend on the algorithm hyperparameters, for each clustering method we have iterated over combinations of relevant sets of hyperparameter values. For the spectral clustering algorithm, we considered the range from two to nine as a possible number of clusters in the data, and 5, 15, 25, 50 as sizes of the neighborhood for computing the affinity matrix. For the precomputed distance matrices of the original data, we use values 1, 5, 10, 15 for the “gamma” parameter in construction of the affinity matrix using a radial basis function. For the PAM algorithm, we used the same range of possible clusters. For the HDBSCAN hyperparameters, we considered 5, 10, 25, 50 as the minimal cluster size and 5, 10, 15, 20 as the minimal number of samples in a neighborhood for a point to be considered a core point. We did not set larger values for the minimal cluster size to avoid conservative clustering when more points will be considered as noise, and clusters will be restricted to more dense areas.

In Figs. 6 and 7, we show the distribution of metrics over all clustering results. They are comprised by partitions calculated for each dataset, taxonomic level, manifold learning method, and clustering algorithm with different hyperparameters. Clustering partitions with moderate or strong support for both metrics correspond to points lying at the intersection of the blue and orange areas. All partitions respective to points in this area were found to consist of two or three highly imbalanced clusters with more than 95% of the data points concentrated in one cluster. These partitions are also inconsistent in terms of clustering validity metrics when either DBCV and Prediction Strength, or Davies–Bouldin and Silhouette pairs pass the thresholds but not all of them. Small clusters that constitute less than 5% of the data were found to be unstable among different partitions of the same dataset, demonstrating high dependence on the manifold learning and clustering algorithms. As expected, different hyperparameters combinations of the clustering algorithm can lead to the same partitions for fixed dataset, taxonomic level and embedding type. Among them, given that they satisfy the clustering metrics, we selected the one with the highest entropy, which should match the most balanced partition.

In Table 5 we present these partitions with the respective metrics. Only few partitions into two and three clusters were found at different taxonomic levels, that meet the threshold criteria of at least one of the metrics pairs: the Davies–Bouldin and Silhouette score or the DBCV and Prediction Strength. All partitions are stable, according to Prediction Strength. Yet, plausible partitions with moderate Silhouette scores varying from 0.60 to 0.74 exhibit low DBCV index from −0.92 to −0.63. Similarly, partitions with moderate DBCV from 0.10 to 0.25 yield lower Silhouette score in the range from −0.02 to 0.23 and higher Davies–Bouldin index varying from 0.87 to 1.38. For all presented clustering partitions, the entropy of the data mass distribution over clusters is low, indicating a highly imbalanced partition. The maximal entropy over two found clusters among partitions is 0.09, indicative of a clustering where 98% of data are concentrated in one cluster. While the entropy of 0.19 for partition into three clusters is higher than for two clusters, it is still imbalanced, with 96% of the data concentrated in the one cluster. Therefore, there are no partitions found, that would satisfy all clustering metrics criteria at the same time.

**Table 5:** Selected clustering results with high or moderate clustering partition metrics. Clustering validity metrics and the number of clusters k for different partitions obtained from different data representations. Repr. denotes a data representation used for clustering. It is either an embedding provided by a manifold learning algorithm (SE - Spectral Embedding, LLE - Locally Linear Embedding) or pairwise distances inferred from the data (L1 - Manhattan distance in the original space of taxonomic abundances). Spectral, Spectral Clustering algorithm. D-B index, Davies-Bouldin index. Silh. score, Silhouette score. DBCV, Density-Based Clustering Validation index. Ent., Entropy. Notation as in Table 2.

	**Tax**	**Repr.**	**Cluster** **method**	**k**	**D-B index**	**Silh. score**	**DBCV**	**Prediction** **Strength**	**Ent.**
**AGP**	**O**	L1	Spectral	2	0.60	0.60	−0.63	0.98	0.06
	**O**	LLE	Spectral	2	0.49	0.74	−0.86	0.94	0.06
	**O**	LLE	Spectral	3	0.60	0.60	−0.91	0.91	0.18
	**O**	SE	Spectral	2	0.50	0.68	−0.91	0.96	0.09
	**O**	SE	Spectral	3	0.57	0.63	−0.92	0.94	0.19
	**F**	t-SNE	HBDSCAN	2	1.38	0.14	0.15	1.00	0.09
	**F**	UMAP	HBDSCAN	2	1.02	0.17	0.22	1.00	0.06
	**G**	UMAP	HBDSCAN	2	1.03	0.23	0.25	1.00	0.08
**HMP**	**O**	t-SNE	HBDSCAN	2	1.00	0.13	0.12	1.00	0.06
	**O**	UMAP	HBDSCAN	2	0.87	0.15	0.19	1.00	0.08
	**O**	UMAP	HBDSCAN	3	1.02	0.06	0.19	1.00	0.16
	**F**	UMAP	HBDSCAN	2	1.03	0.08	0.10	1.00	0.08
	**F**	SE	HBDSCAN	2	0.53	0.64	−0.63	1.00	0.09
	**F**	t-SNE	HBDSCAN	2	1.11	0.09	0.21	0.97	0.09
	**G**	UMAP	HBDSCAN	2	1.24	−0.02	0.16	1.00	0.06

**Figure 8:**
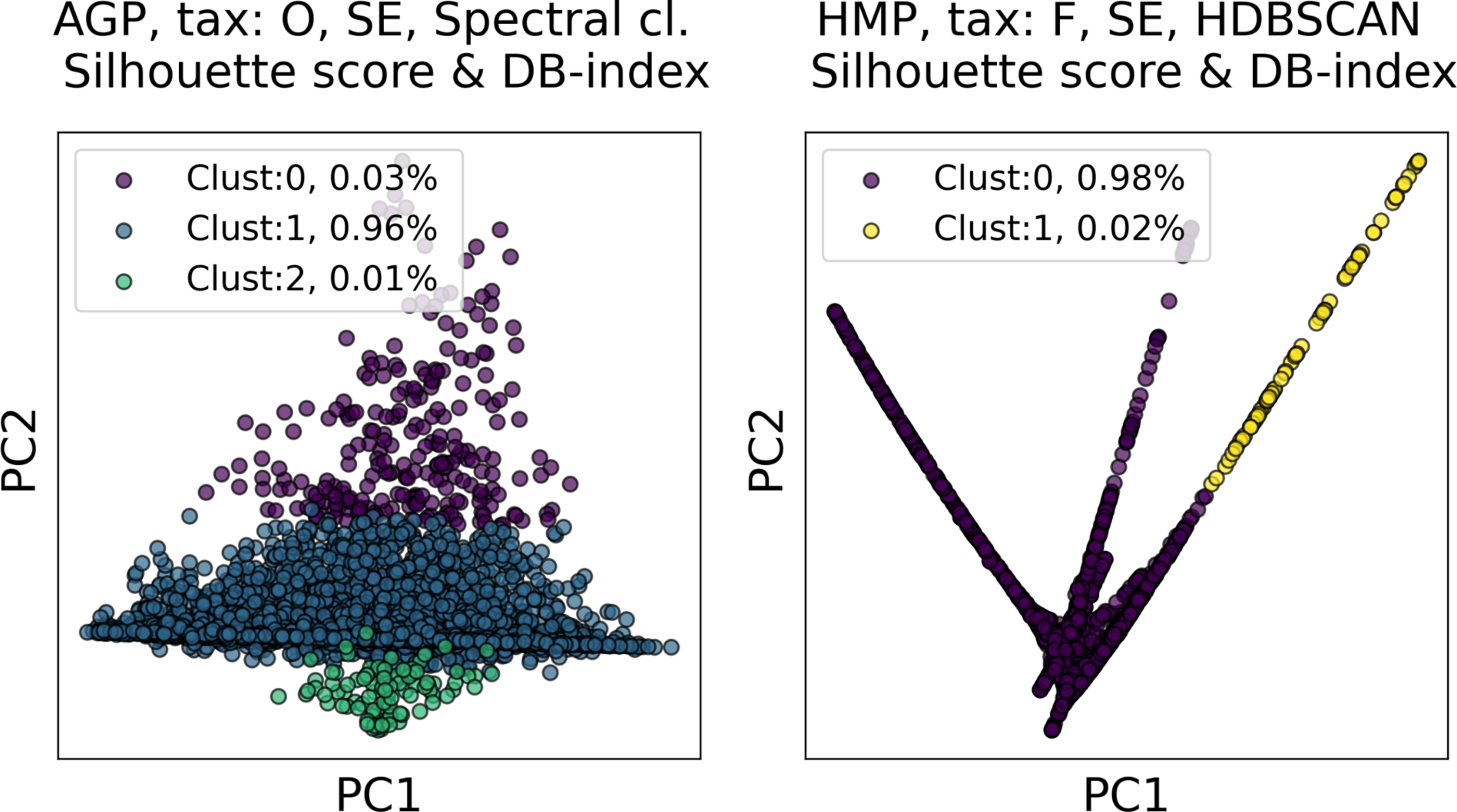
Visualization of the clustering results for the AGP and HMP datasets in the first two principal components. The visualized clustering partitions have the highest entropy among all other partitions satisfying Silhouette score and Davies–Bouldin (DB) Index thresholds. Dataset name, taxonomy level, representation of the data, clustering algorithm, and the pair of metrics used to select the partition are shown in the title. SE, spectral embedding. Color indicates different clusters. The percentage of the data belonging to each cluster is depicted on the legend.

**Figure 9:**
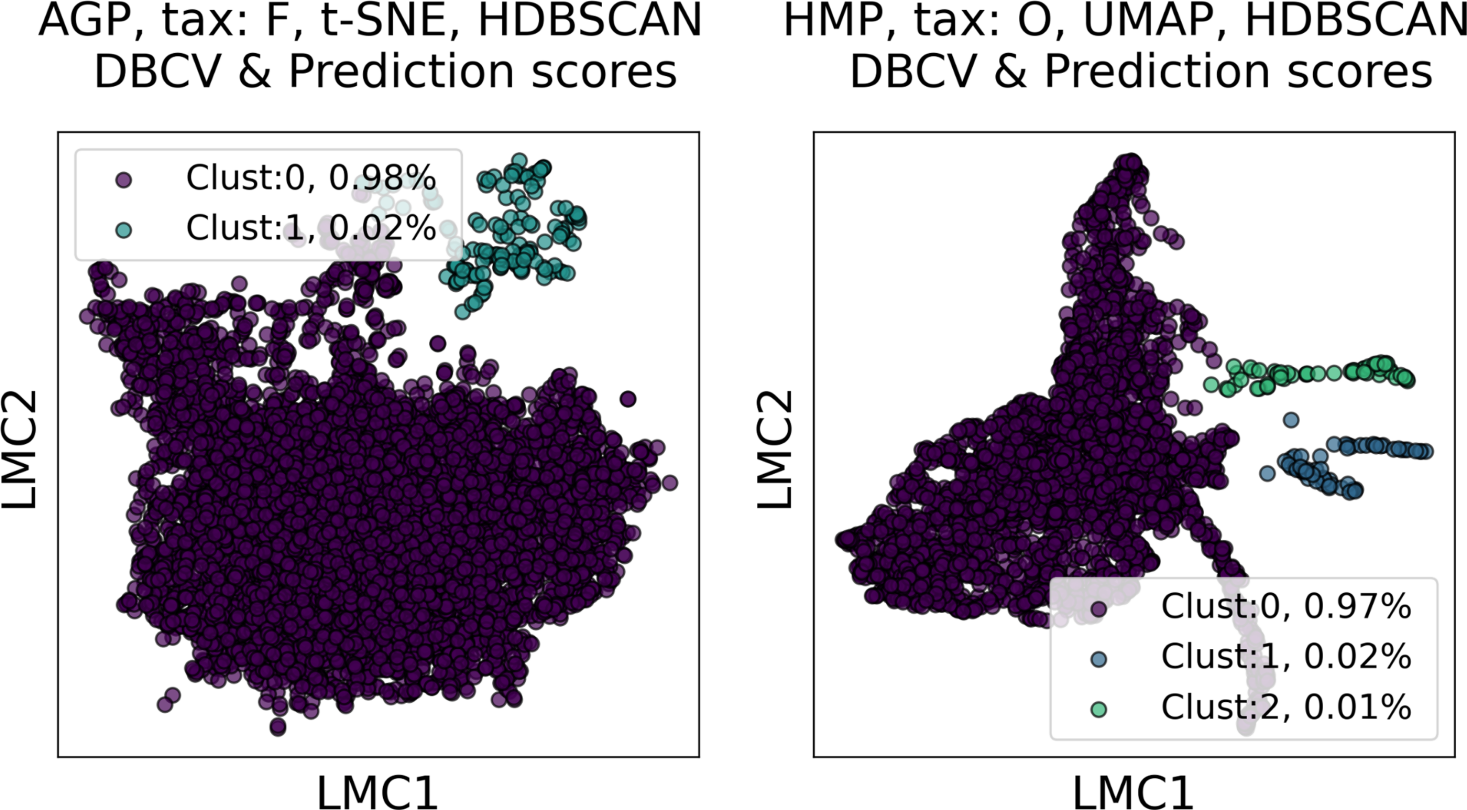
Visualization of the clustering results for the AGP and HMP datasets, using Large Margin Nearest Neighbor method. The visualized clustering partitions have the highest entropy among all partitions satisfying DBCV index and prediction strength thresholds. Dataset name, taxonomy level, representation of the data, clustering algorithm, and the pair of metrics used to select the partition are shown in the title. Color indicates different clusters. The percentage of the data belonging to each cluster is depicted on the legend.

Among all clustering partitions passing the metrics thresholds in Figs. 6 and 7, we visualize the ones with the highest entropy. The resulting projections for AGP and HMP datasets, for every pair of metrics, are presented in Figs. 8 and 9. Since these partitions were found in an embedding space with dimensionality larger than three, we use for visualization PCA in Fig. 8 and Large Margin Nearest Neighbor method (Weinberger & Saul, 2009) in Fig. 9. Different approaches were chosen for the sake of better visualization of the clustering partition. The large margin nearest neighbor method allows for dimensionality reduction *via* linear transformation. This transformation is conditioned on the clustering partition of the data so that neighbor points from the same cluster are kept close, whereas points from different clusters are separated by a large gap.

Further, we have demonstrated that straightforward assignment of each data point to a potential enterotype, based on the originally reported distribution of *Bacteroides*, *Prevotella* and *Ruminococcus* Genera in enterotypes (Arumugam et al., 2011), does not reveal any natural clusters in different data representations. The corresponding distributions of clustering metrics and the visualization are presented in Text S4, Figs. S6–S7.

Together, these results imply that various clustering methods along with different manifold learning algorithms yield only highly imbalanced partitions, with more than 95% of data concentrated in one cluster. We do not consider clusters that contain less than 5% of the data as enterotypes. We attribute these small clusters to artifacts of manifold learning algorithms since there is evidence (Cooley et al., 2019; Kobak & Berens, 2019) that common dimensionality reduction techniques may fail to faithfully represent the original point cloud distribution, introducing substantial distortion into the data. We observe that these small clusters are not stable, depending on the clustering method and the manifold learning algorithm. Hence, the stool metagenomes can hardly be divided into stable and distinct clusters that could be referred to as enterotypes. Our simulation on a synthetic dataset, presented in Text S2, Table S1, and Fig. S1, proves that distinct and stable clusters related to enterotypes have not been found because of their absence in the data rather than methodology flaws.

### Visualization

**Figure 10:**
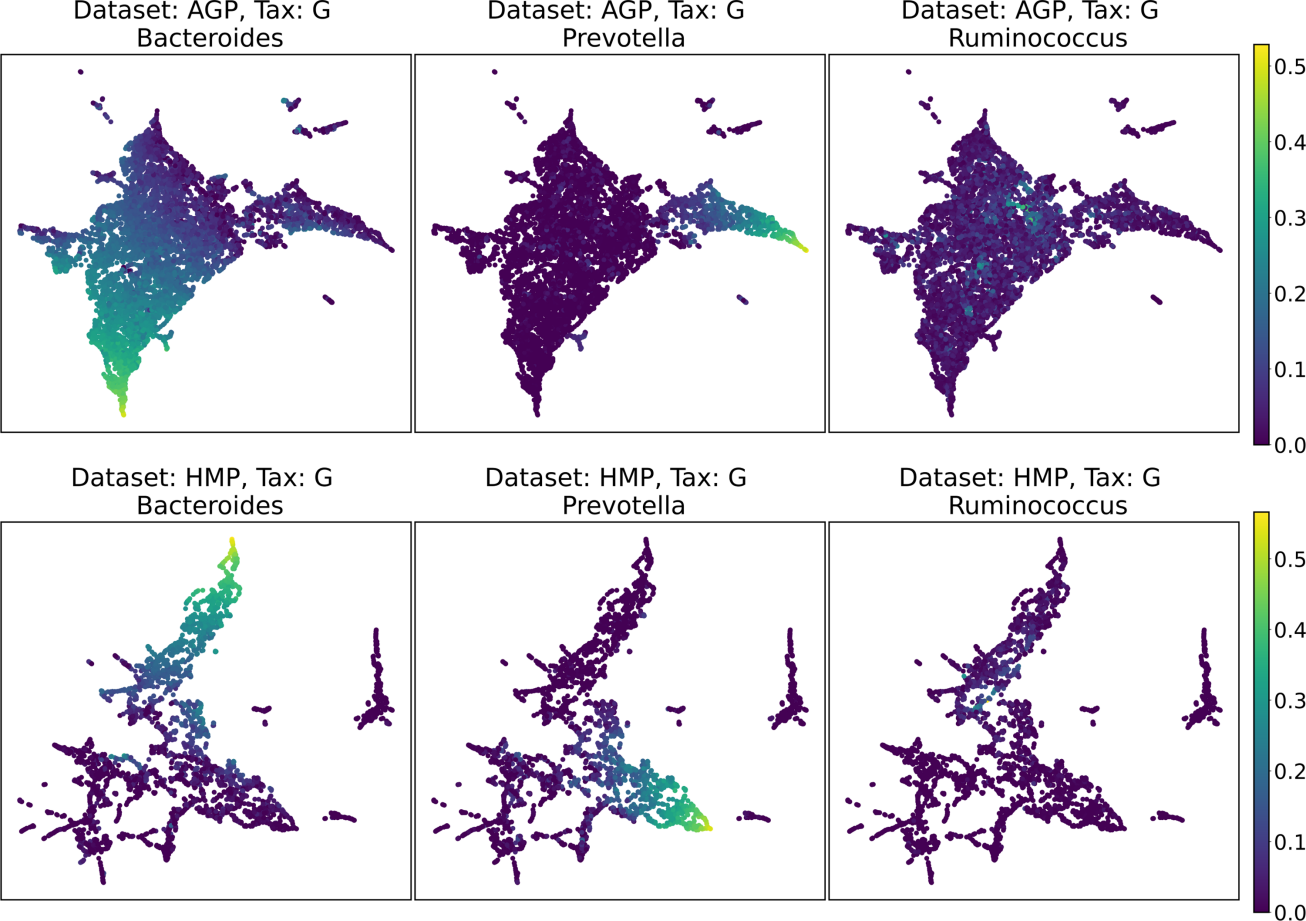
2D UMAP visualization of AGP and HMP datasets for the Genus taxonomy level. Colors reflect the relative abundance of specific taxa, see the headers.

**Figure 11:**
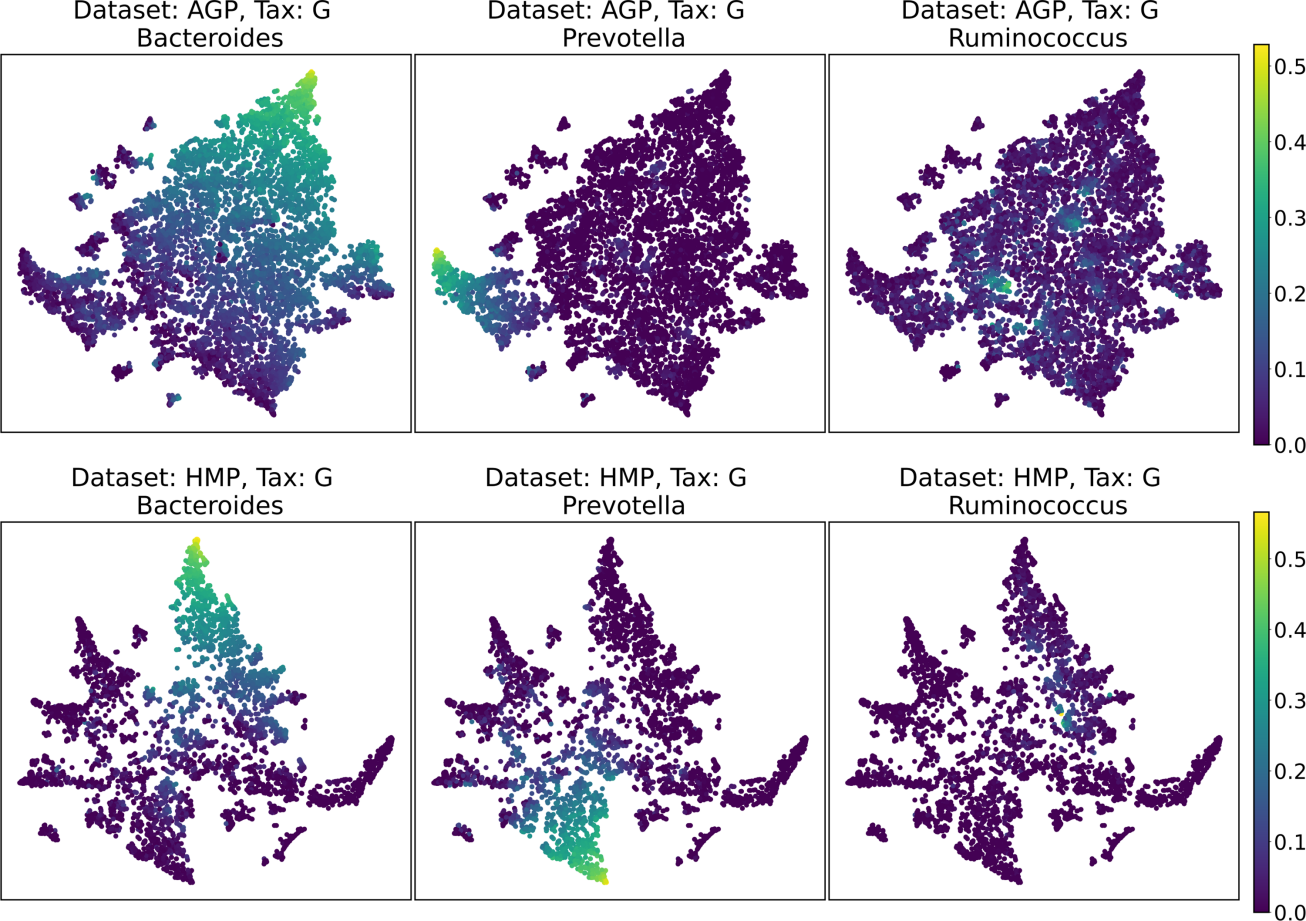
2D t-SNE visualization of AGP and HMP datasets for the Genus taxonomy level. Colors reflect the relative abundance of specific taxa, see the headers.

Despite the lack of distinct and stable clusters in the data, we demonstrate that human gut microbial communities vary continuously along a low-dimensional manifold. We observe the structure of such a manifold by mapping the point clouds of data from the Genus taxonomic level on a two-dimensional plane using UMAP in Fig. 10 and t-SNE in Fig. 11. As explained above, prior to this step the datasets have been projected on the principal components capturing 99% of variance. To remove noise and outliers, after the dimensionality reduction small clusters of points containing less than 1% of the data were removed using the Local Outlier Factor algorithm (Breunig et al., 2000). To demonstrate the continuity of the data points distribution, we colored points as specific taxon relative abundances, corresponding to the genera most relevant for the definition of enterotypes, according to the initial finding (Arumugam et al., 2011). Salient parts of the manifold represent higher concentrations of specific OTUs. Small clusters observed in Figs. 10 and 11 are not related to enterotypes, being the direct result of specific methods hyperparameters, that may lead to tearing off the salient part of the data manifold.

**Figure 12:**
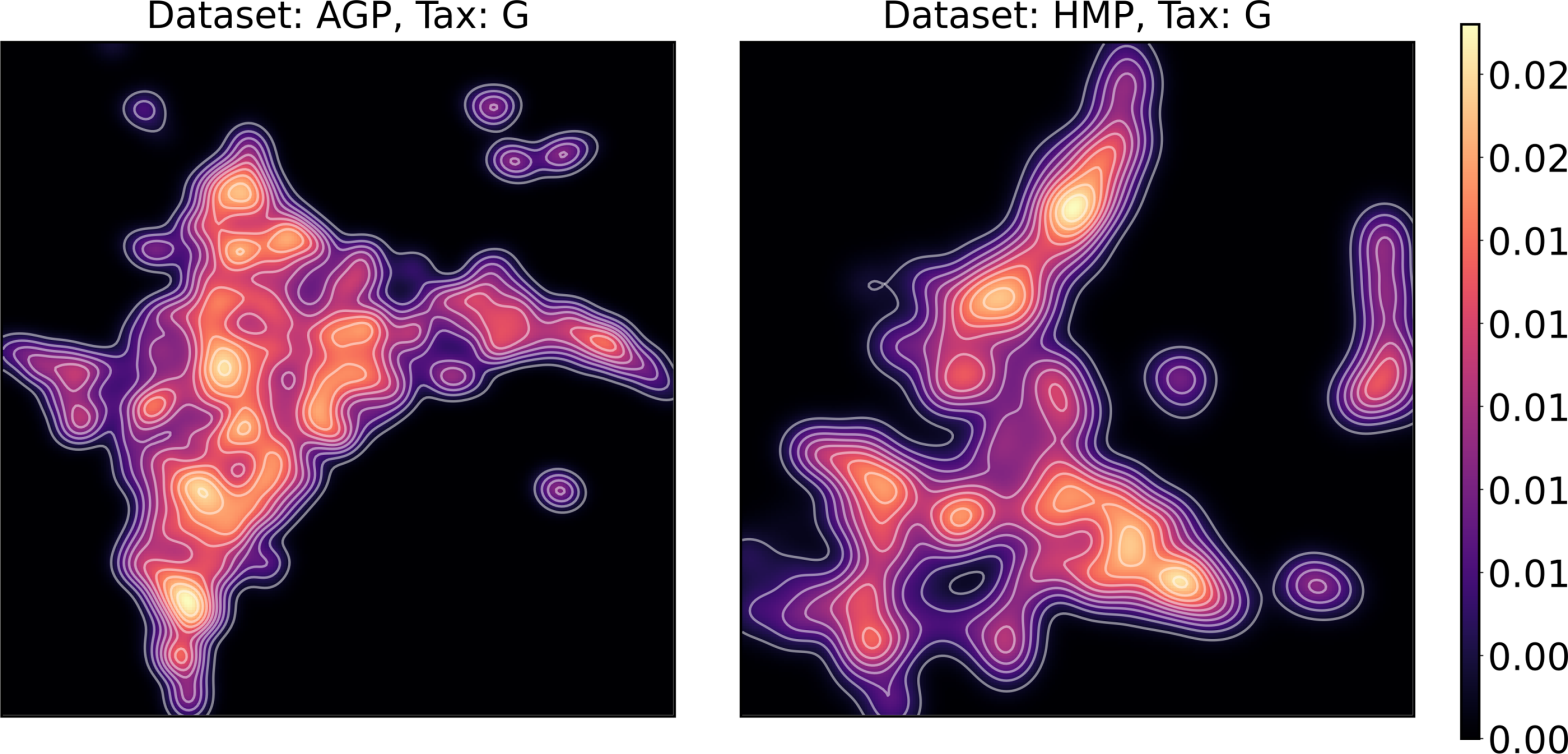
Kernel density estimation (KDE) of 2D UMAP visualization. Color indicates relative likelihood of the point to belong to the data distribution, according to KDE.

**Figure 13:**
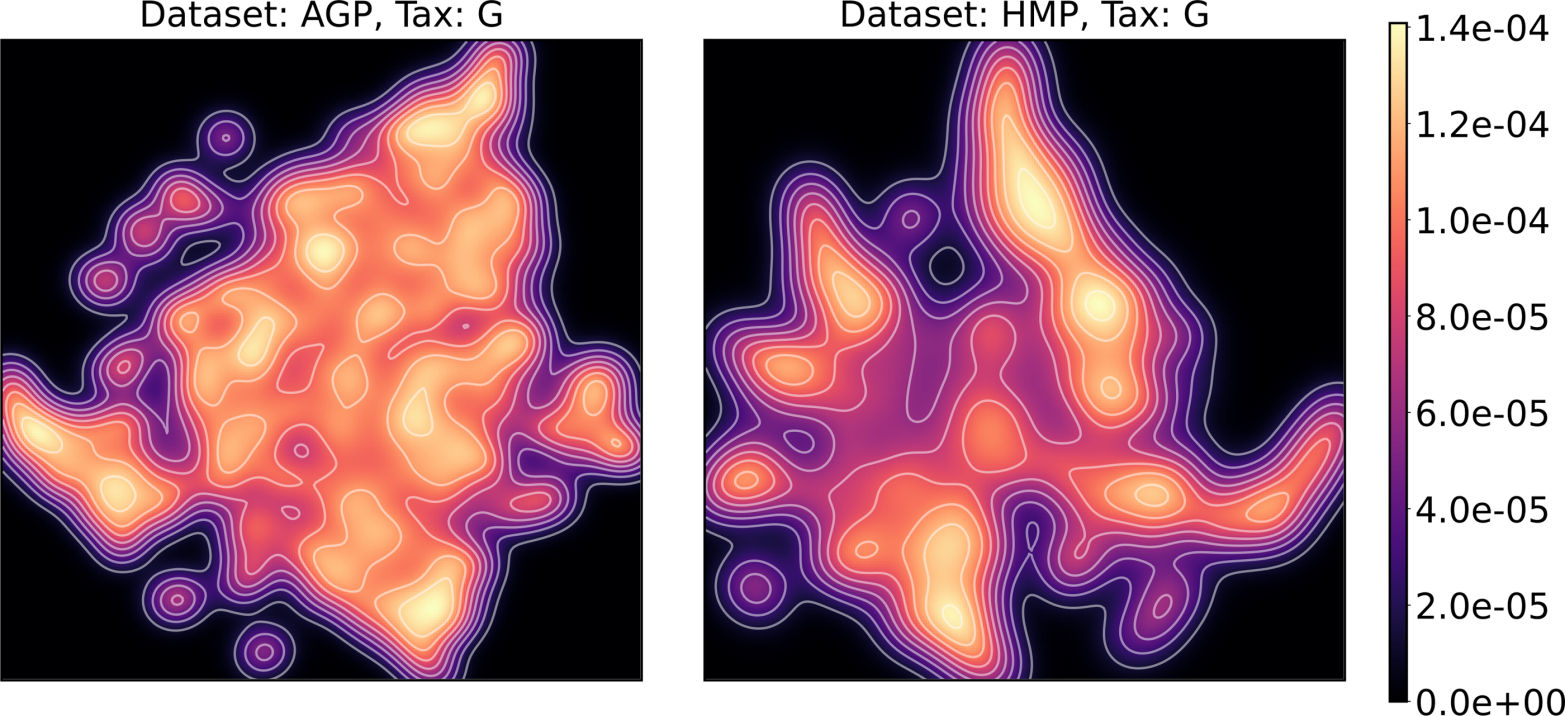
Kernel density estimation (KDE) of 2D t-SNE visualization. Color indicates relative likelihood of the point to belong to the data distribution, according to KDE.

To estimate the density of points in the visualization, we performed standard kernel density estimation (KDE) of this 2-dimensional data. The bandwidth parameter of the KDE is equal to the median value of pairwise distances distribution from every point to 100 closest neighbors. For the two-dimensional visualizations produced by both UMAP and t-SNE in Figs. 10 and 11, we observe that the density of data distribution is not uniform, indicating that there are regions of preferential concentrations of data, as shown in Fig. 12 for UMAP and in Fig. 13 for t-SNE algorithm. Nevertheless, this can be related only to the variations of OTU concentration (*Bacteroides* and *Prevotella*) and features of the dimensionality reduction methods rather than to formation of distinct clusters. This is ensured by the density-based clustering algorithm HDBSCAN, robust to noise and clusters shape, that would have indicated the existence of clusters by the high DBCV metric. Therefore, these regions are not related to the enterotypes in the original definition.

**Figure 14:**
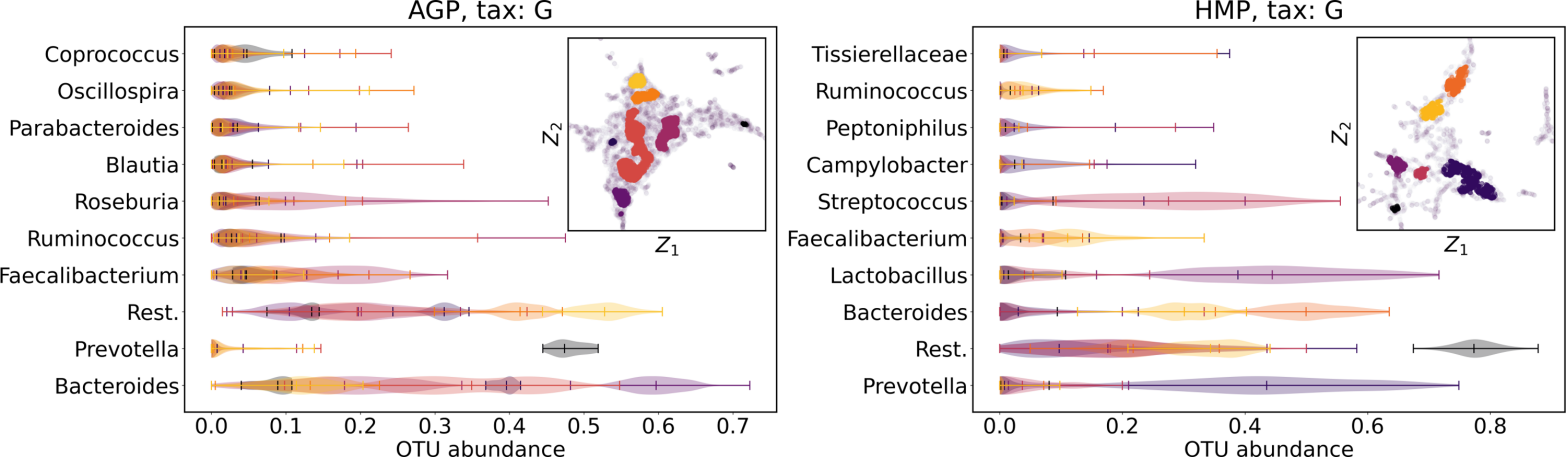
Analysis of the high-density regions of 2D UMAP visualization. The colored regions correspond to the kernel density estimation likelihood larger than 70% percentile of the total likelihood distribution. Color indicates different high-density clusters, depicted in the two-dimensional scatter plot with Z_1_ and Z_2_ coordinates. For the first ten selected OTU with the largest mean value among all high-density regions, the violin plots depict their distribution within each region.

**Figure 15:**
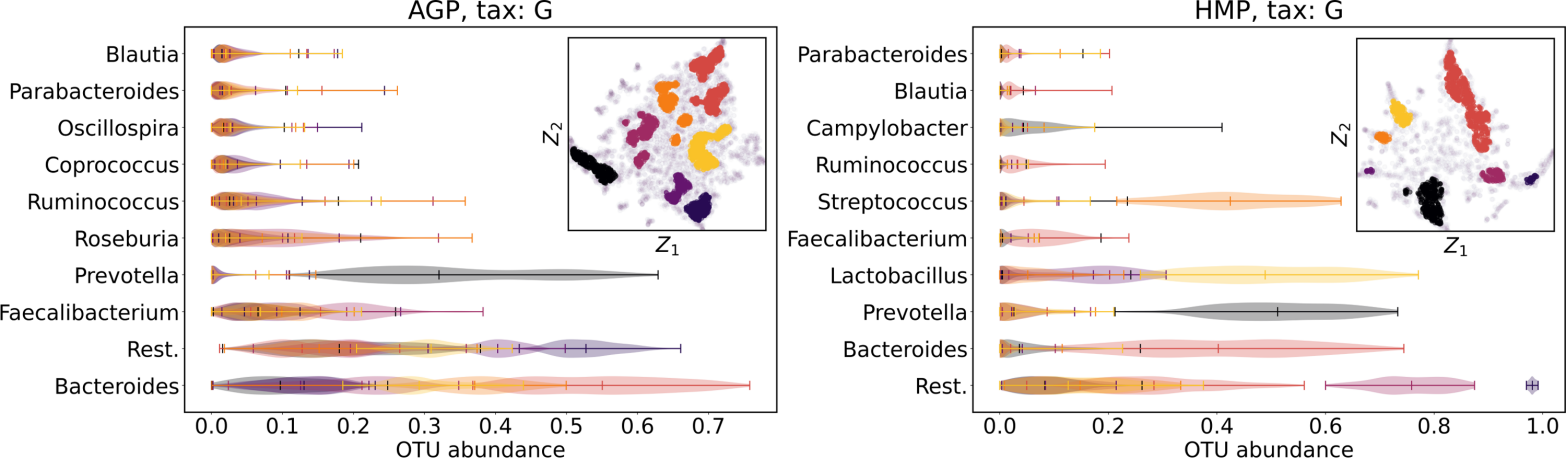
Analysis of the high-density regions of 2D t-SNE visualization. The colored regions correspond to the Kernel Density Estimation likelihood larger than 70% percentile of the total likelihood distribution. Color indicates different high-density clusters, depicted in the two-dimensional scatter plot with Z_1_ and Z_2_ coordinates. For the first ten selected OTU with the largest mean value among all high-density regions, the violin plots depict their distribution within each region.

We supported this intuition by analyzing the OTU distribution in the high-density areas of visualizations in Figs. 10 and 11. Removing all regions in Figs. 12 and 13 with density less than 70% percentile of the total density distribution, we obtained separated, high-density areas. In Figs. 14 and 15, we show these regions in the two-dimensional visualization, along with the distributions of the ten most significant OTUs within the regions. The most significant OTUs are the ones with the largest mean value among all points that belong to the high-density regions. We observe that for the UMAP visualization in Fig. 14, the difference in the OTU distribution between clusters is mostly controlled by *Bacteroides* and *Prevotella* and an unclassified OTU at the Genus level, denoted as *Rest*. This observation is consistent with the abundance gradient visualization in Figs. 10 and 11, as well as previously reported results (Costea et al., 2017), indicating the continuous nature of the OTU distribution with preferential high-density regions. The same applies to the analysis of the t-SNE visualization in Fig. 15, with the difference, that the variation between high-density regions is also controlled by *Faecalibacterium* for AGP and *Lactobacillus and Streptococcus* for HMP.

## Discussion

Our results demonstrate that the metagenome distribution is continuous rather than discrete and lies on a low-dimensional non-linear manifold embedded in the original high-dimensional space of relative taxon abundances. We posit that most of the previous observations may have been artifacts caused by limitations of linear methods applied for the analysis of non-linear, high-dimensional metagenomic data. Also, overfitting in the data density estimation may occur due to insufficient numbers of data points. Small sizes of datasets lead to unstable clustering, especially if the latter is performed in a high-dimensional space. One may suggest an intuitive explanation of why positive clustering results were widespread in previous works. In most of them, small datasets were used, which makes the total number of intermediate microbial patterns negligible. We suppose that datasets demonstrating moderate clustering in related works have been sampled from high-density areas in the general taxonomic abundance space. A more discrete structure could arise if more diverse samples are studied, including people with sharply differing lifestyles and diets. Still, successful attempts to correct the human gut microbiota were made, *e.g.*, fecal microbiota transplantation to treat the *Clostridium difficile* infection (CDI) (Smits et al., 2013), inflammatory bowel disease (IBD) (Suskind et al., 2015), and obesity (Vrieze et al., 2012). Connecting the distribution of microbiome abundances and structural features of the microbial manifold with lifestyle, nutrition, or disease may be a promising direction for further research.

## Conclusions

We have shown the absence of enterotypes, defined as stable dense regions, or as stable and well separated clusters in the taxonomic abundance space, in human gut microbiomes. This challenges the current consensus, demonstrating that the metagenome distribution is continuous rather than discrete. We improved the standard methodology of microbial data analysis by applying a large variety of linear and non-linear dimensionality reduction methods to properly estimate the intrinsic dimension. We demonstrate that some of these methods do preserve the global and local data structure. This allowed us to achieve robustness of the clustering methods and compare results produced by different approaches. To the best of our knowledge, this is the first study applying a wide range of non-linear methods for validating the existence of enterotypes, and hence it may serve as a starting point for a more adequate analysis of metagenome datasets, which may reveal an intrinsic connection to nutrition, lifestyle, or disease. This study is also relevant for computational biologists seeking a general approach for clustering in high-dimensional data.

##  Supplemental Information

10.7717/peerj.15838/supp-1 Supplemental Information 1 Supplemental information

## Additional Information and Declarations

### Data Availability

The following information was supplied regarding data availability: Data and software are available at Figshare: Bulygin, Ivan; Shatov, Vladislav; Rykachevskiy, Anton; Raiko, Arsenii; Bernstein, Alexander; Burnaev, Evgeny; et al. (2022). Absence of enterotypes in the human gut microbiomes reanalyzed with non-linear dimensionality reduction methods. figshare. Dataset. https://doi.org/10.6084/m9.figshare.19091423.v5
The software is also available at GitHub: https://github.com/blufzzz/Human-Gut-Microbiome-Analysis.
