# Supplementary material for: Absence of enterotypes in the human gut microbiomes reanalyzed with non-linear dimensionality reduction methods
Source: PeerJ. 2023 Sep 8;11:e15838. doi: 10.7717/peerj.15838 (PMC10494839; doi:10.7717/peerj.15838)
Supplement: Supplemental Information 1 [file peerj-11-15838-s001.pdf]

Supporting Information of
Absence of enterotypes in the human gut microbiomes reanalyzed with non-linear dimensionality reduction methods

Ivan Bulygin[1,*], Vladislav Shatov[2], Anton Rykachevskiy[1], Arsenii Raiko[1], Alexander Bernstein[1], Evgeny Burnaev[1,3], and Mikhail S. Gelfand[1,4]

[1] *Skolkovo Institute of Science and Technology, Moscow, 121205, Russia*
[2] *School of Biology, Moscow State University, Moscow, 119991, Russia*
[3] *Artificial Intelligence Research Institute (AIRI), Moscow, Russia*
[4] *Institute for Information Transmission Problems (the Kharkevich Institute, RAS), Moscow, 127051, Russia*
[*] *Corresponding author: bulygin@phystech.edu*

Text S1 Metrics

The Silhouette score [10] shows how well clusters are separated, by calculating the average difference between the similarity of a data point to its own cluster compared to the similarity to other clusters, with higher values indicating better clustering. For a data point i, Silhouette score S_i is defined as:

$$S_i = \frac{(b_i - a_i)}{\max(b_i, a_i)} \tag{1}$$

In Eq. 1, a_i is the mean intra-cluster distance and b_i is the mean distance between the sample point i and the data points from the nearest cluster C_k that sample i is not a part of.

$$a_i = \frac{1}{|C_i| - 1} \sum_{j \in |C_i|, i \neq j} d(i, j) \tag{2}$$

$$b_i = \min_{k \neq i} \frac{1}{|C_k|} \sum_{j \in |C_k|} d(i, j) \tag{3}$$

Here in Eq. 2, 3, $|C_i|$ is the number of points in a cluster C_i and $d(i, j)$ is the distance between data points i and j. The total Silhouette score is measured as the mean of all S_i and bounded between -1 for incorrect clustering and +1 for dense and well-separated clusters.

The Davies-Bouldin index DB [3] in Eq. 4 measures the average similarity of the distance between clusters with the size of the clusters themselves. It estimates the cohesion based on the distance from the points in a cluster to its centroid and the separation based on the distance between centroids. Values closer to zero indicate better clustering, whereas higher values indicate overlapping partitions.

$$DB = \frac{1}{K} \sum_{i=1}^{K} \max_{j: j \neq i} R_{ij} \qquad R_{ij} = \frac{s_i + s_j}{d(\mu_i, \mu_j)} \tag{4}$$

In Eq. 4, s_i is the average distance between each point of cluster i and a centroid μ_i of that cluster, K is the total number of clusters, and $d(\mu_i, \mu_j)$ is the distance between centroids μ_i and μ_j of the clusters i and j.

Both Silhouette score and Davies-Bouldin index are relatively easy to compute, but they are generally higher for convex clusters than for the density based ones. In the presence of the non-convex clusters and noise, they may fail to indicate the presence of a valid clustering partition. Density-Based Clustering Validation was proposed as a metric for assessing the clustering partition quality for detecting non-convex clusters in the presence of noise. The metric is based on the Hartigan model of Density Contour Trees [4] and provides values between -1 and +1, with greater values indicating a better density-based clustering solution. See [8] for details.

To estimate the stability of the data X partition $C(X,k)$ into k clusters, we use Prediction Strength ps(k), see [12]. Following the m-fold cross-validation technique, we split m times the data X into X_{tr} and X_{te} with sizes n_{tr} and n_{te}, such that all obtained X_{te} will cover the X. For each such split, we estimate the ps(k) as in Eq. 5 and then average all of them across the splits. In Eq. 5, k is the number of clusters, $A_{k1}, A_{k2}, ...A_{kk}$ are the indices of the test clusters $1, 2, ...k$ with corresponding sizes $n_{k1}, n_{k2}, ...n_{kk}$. They were obtained from clustering the test data X_{te} into k clusters - $C(X_{\text{te}}, k)$. Taking into account clustering decision boundaries from X_{tr} data, the $D[C(X_{\text{tr}}, k), X_{\text{te}}]$ is $n_{te} \times n_{te}$ matrix, with its ii' element equal to one if the observations i and i' from X_{te} fall into the same cluster, and zero otherwise. To construct $D[C(X_{\text{tr}}, k), X_{\text{te}}]$, we estimate the clustering decision boundaries for X_{te} from $C(X_{\text{tr}}, k)$ using k-Nearest Neighbour classification with distance weighting, Euclidean metric and five nearest neighbours.

$$\text{ps}(k) = \min_{1\leq j\leq k} \frac{1}{n_{kj}(n_{kj}-1)} \sum_{i\neq i'\in A_{kj}} D[C(X_{\text{tr}}, k), X_{\text{te}}]_{ii'} \tag{5}$$

To assess the disbalance in a clustering partition, we use the Shannon Entropy [11] H of the distribution that indicates the probability p_i of one of n data points to fall into a cluster i with $|C_i|$ data points in it, see Eq. 6. Higher H values indicate more balanced partition - when we are less sure in which cluster point is residing.

$$H = -\sum_{i=1}^{k} p_i log(p_i) \qquad p_i = \frac{|C_i|}{n} \tag{6}$$

We estimate the reconstruction error of inverse mapping $\hat{x}_i = g(z_i)$ from the manifold learning embedding $z \in Z$ to the original space of relative taxon abundances as the normalized Median Absolute Error as in Eq. 7:

$$\text{MAE}(X, \hat{X}) = \text{median}\{\frac{|x_i - \hat{x}_i|_1}{|x_i|_1}, i \in \overline{1, N}\} \tag{7}$$

Selecting and supervised training of the inverse mapping algorithm g brings ambiguity into the method. To mitigate it, we use additional scale-independent rank-based quality criteria for dimensionality reduction proposed in [7]. Given a distance metric $d(i,j)$, two rank matrices are constructed $\rho_{ij} = \{|k : d(x_k, x_j) < d(x_i, x_j)|\}$ for the original data points $x_i \in X$ and $r_{ij} = \{|k : d(z_k, z_j) < d(z_i, z_j)|\}$ for their low-dimensional embeddings $z_i \in Z$. Then, a co-ranking matrix Q is built, where $Q_{kl} = \{|(i,j) : \rho_{ij} = k,\ r_{ij} = l|\}$ indicates the total number of instances when the k-th neighbor for some point became l-th. Thus, all non-diagonal elements of this matrix correspond to changes in arrangement of the embedded points compared to the original data. The co-ranking matrix contains all information on how the data structure is distorted in a low-dimensional representation. This information is represented further as two scalars Q_{loc} and Q_{glob} that range from 0 (bad) to 1 (good) and reflect the preservation of the "local" and "global" structure of the data cloud. The Q_{loc} and Q_{glob} encompass most of the co-ranking metrics properties, mitigating our need for a unified model-free dimensionality reduction criteria since each manifold algorithm we are using is defined with unique quality criteria. See [7] for details.

For similarity measure between the predicted clustering partition X and given ground-truth labels Y we utilize Adjusted Rand Index [5], based on the Rand Index (RI) [9]. Given the predicted $X = \{X_i\}_{i=1}^{r}$ and the true $Y = \{Y_j\}_{j=1}^{s}$ clustering partitions into r and s clusters respectively, the Rand Index is non-zero for random independent partitions X and Y and 1 for the same partitions up to labeling permutations. The Adjusted Rand Index is "adjusted" version of RI such its values are close to 0 for random labeling. In Tab. 8 a contingency table is presented, where n_{ij} denotes the common number of points in clusters X_i and $Y_j : n_{ij} = |X_i \cap Y_j|$. Given a_i, b_j, n_{ij} from Tab. 8, the Adjusted Rand Index (ARI) is defined as Eq. 9. The $C_n^k = \frac{n!}{k!(n-k)!}$ denotes a k-combination from a set of n elements.

X	Y_1	Y_2	$\cdots$	Y_s	$a_i = \sum_k n_{ik}$
X_1	n_{11}	n_{12}	$\cdots$	n_{1s}	a_1
X_2	n_{21}	n_{22}	$\cdots$	n_{2s}	a_2
$\vdots$	$\vdots$	$\vdots$	$\ddots$	$\vdots$	$\vdots$
X_r	n_{r1}	n_{r2}	$\cdots$	n_{rs}	a_r
$b_j = \sum_k n_{kj}$	b_1	b_2	$\cdots$	b_s	

(8)

$$\text{ARI} = \frac{\sum_{ij} C_{n_{ij}}^2 - \left[\sum_i C_{a_i}^2 \sum_j C_{b_j}^2\right]/C_n^2}{\frac{1}{2}\left[\sum_i C_{a_i}^2 + \sum_j C_{b_j}^2\right] - \left[\sum_i C_{a_i}^2 \sum_j C_{b_j}^2\right]/C_n^2} \tag{9}$$

Text S2 Synthetic dataset

In this section, we provide the results of our method on synthetic data. The positive control datasets with clusters were created similarly to [6]. We generated nine synthetic datasets with the number of clusters k ranging from two to four, with different dimensionality d similar to the O,F and G taxonomy levels, if we remove OTU found in less than 1% of the samples or with a standard deviation less than 0.001. The dimensionalities are 39, 70 and 108 correspondingly. Each dataset consists of 3000 data points representing vectors of un-normalized abundances of mock OTUs. 90% of data points were sampled from one of k multivariate gaussian distributions, each reflecting a cluster, and other 10% are sampled from a distribution with a larger variance, representing noise. To compare estimated clustering partitions with the ground-truth labels, we used the Adjusted Rand Index [5] (see Supplementary Notes Text S1 for details). The dimensionality d_{PCA} that retains 99% of the cumulative explained variance after the PCA projection, estimated intrinsic dimension, the Median Absolute Error (MAE) and Q_{loc}, Q_{glob} metrics are reported in Tab. S1.

d	k	d_{PCA}	d_{MLE}	MAE	Q_{loc}	Q_{glob}
39	2	38	22	0.052	0.87	0.98
39	3	38	21	0.049	0.88	0.99
39	4	38	20	0.047	0.88	0.99
70	2	68	31	0.070	0.87	0.98
70	3	68	29	0.063	0.87	0.98
70	4	67	28	0.075	0.87	0.98
108	2	104	39	0.084	0.85	0.98
108	3	103	37	0.087	0.85	0.98
108	4	103	36	0.087	0.86	0.98

Table S1: Original dimensionality d, number of clusters k, dimensionality d_{PCA} after PCA projection and estimated intrinsic dimensionality d_{MLE} of synthetic datasets. For each dataset MAE, Q_{loc} and Q_{glob} metrics were computed after the PCA projection with respect to the original data.

In Fig. S1 we display the clustering metrics distribution over all partitions that have been calculated for each dataset with a given number of clusters k and dimensionality d, each manifold learning method, and each clustering algorithm with a different combination of its hyperparameters, described in the Materials & Methods section of the manuscript. Clustering partitions with moderate or strong support for both metrics correspond to points lying at the intersection of the blue and orange areas in Fig. S1. For each dataset the most accurate clustering partition was found in the UMAP embedding by the HDBSCAN algorithm. Such partitions have the Adjusted Rand Index and the Prediction Strength higher than 0.99, the Davies-Bouldin index lower than 0.3, the Silhouette score higher than 0.8 and DBCV higher than 0.8. Moreover each such clustering exhibit Entropy over 0.6, which corresponds to the balanced partition. Together, these metrics assert the presence of stable and distinct clusters in the data for every number of clusters k and dimensionality d.

Text S3 Original datasets

In the following section we provide clustering analysis results for the Sanger, Illumina and Pyroseq (pyrosequencing-based 16S RNA) datasets from the original studies [1], as mentioned in the Materials & Methods section of the manuscript. The sizes of the datasets are 33, 85 and 154 samples, and the dimensionalities at the Genus taxonomy level are, respectively, 249, 483 and 165. Since the number of dimensions, relative to the number of samples, is too large for each dataset, application of the dimensionality reduction methods is unreasonable due to the insufficient amount of data. Therefore, we skipped the estimation of the intrinsic dimension and manifold learning steps for these datasets. Still, we retained preprocessing step with normalization by dividing the Operational taxonomy Units (OTUs) values by the total sum of abundances for a given data sample. For the PCA step we assess number d_{PCA} of the principal components retaining 99% of the cumulative variance. We estimate the Median Absolute Error (MAE) and Q_{loc}, Q_{glob} metrics of the linear dimensionality reduction by projection on principal components. These metrics are presented in Tab. S2. The sorted cumulative sum of the singular values for d_{PCA} estimation is presented in Fig. S2 along with the PCA loadings. These loadings indicate contribution of the OTU coordinates to the principal components. As one can see, the abundances of *Prevotella* and *Bacteroides* genera along with *Lachnospiraceae* Family (includes *Roseburia* and *Blautia* Genus) contribute most to the principal components coordinates.

We applied the original clustering approach that had initially revealed enterotypes in [1]. It exploits the Partition Around Medoids (PAM) algorithm with the Jensen-Shannon distances estimated in the original space of normalized taxonomy abundances. Following this approach, we did not include the unassigned fraction of metagenomic reads

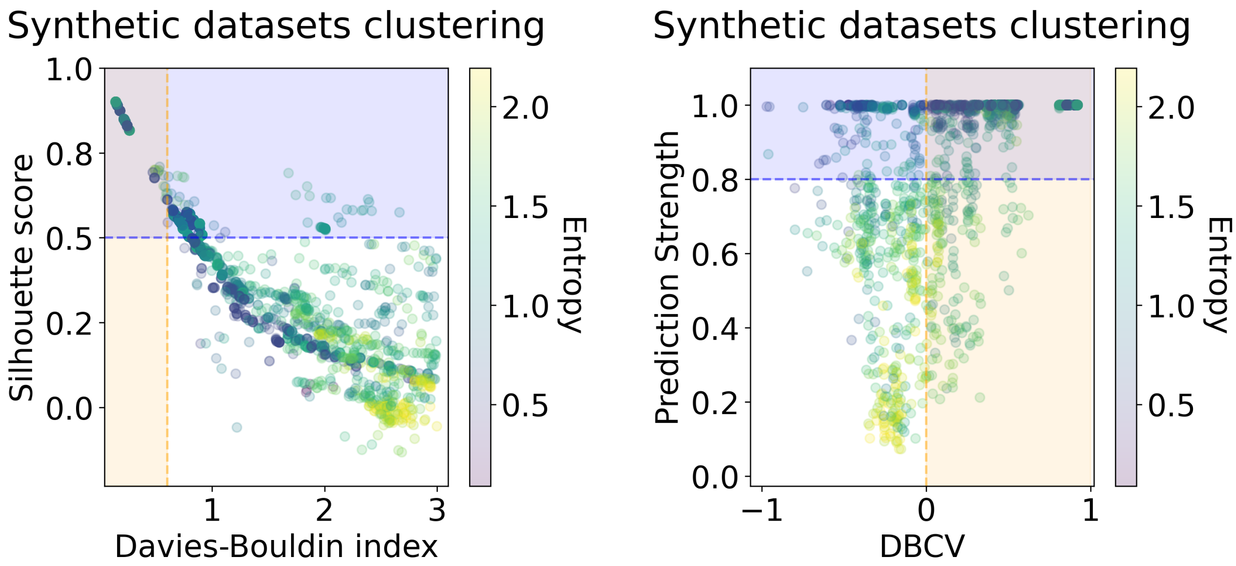

Figure S1: Silhouette score and Davies-Bouldin index (left), DBCV index and Prediction Strength (right) of clustering partitions for all nine synthetic datasets with the number of clusters k equal to 2, 3 and 4, and the dimensionality d equal to 39, 70 and 108.

Dataset	Size	$d_{\text{init.}}$	$d_{\text{proc.}}$	d_{PCA}	MAE	Q_{loc}	Q_{glob}
Sanger	33	249	34	13	0.141	1.00	0.99
Illumina	85	483	27	11	0.136	0.87	0.98
Pyroseq	154	165	58	20	0.141	0.92	0.99

Table S2: Original dimensionality $d_{\text{init.}}$, dimensionality after preprocessing $d_{\text{proc.}}$ and after PCA projection d_{PCA}. For each dataset the Median Absolute Error (MAE), Q_{loc}, and Q_{glob} metrics were computed after the PCA projection with respect to the preprocessed data.

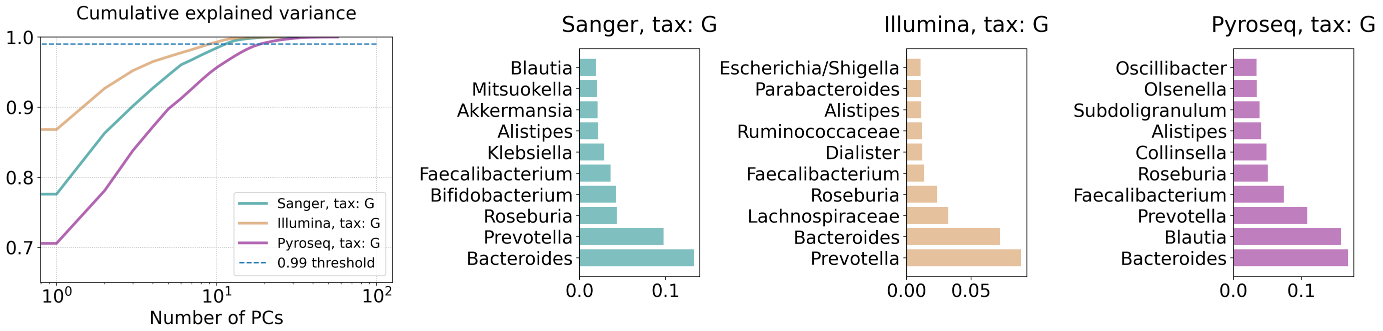

Figure S2: Cumulative explained variance of the Principal Component Analysis (PCA) and PCA loadings, representing contribution of the original taxonomy coordinates to the principal components.

as a feature while estimating the distances. As a result, we obtain reference clustering partitions, shown in Fig. S3. We compare them to the selected clustering partitions obtained within our framework are presented in Fig. S5. As a similarity measure between different partitions of the same data, we use Adjusted Rand Index. Due to the application of a wider range of clustering methods and diverse data representations, our framework revealed clustering partitions with substantially better metrics. Ideally, partitions in our approach are represented as points lying in the overlap of the orange and blue areas in Fig. S4. Nevertheless, according to the Silhouette score and the Davies-Bouldin index there are no clusters that are distinct and balanced at the same time. The only distinct partition that passes the Silhouette score and the Davies-Bouldin index thresholds was found for the Illumina dataset in Fig. S4 (top). Yet this partition is unbalanced, as indicated by the low entropy value. Most partitions were identified by the DBCV and Prediction Strength metrics for Sanger and Illumina datasets in Fig. S4 (bottom). For every pair of metrics for each dataset, we find the best partition and visualize them in Fig. S5. The best partition is chosen either by maximizing entropy among

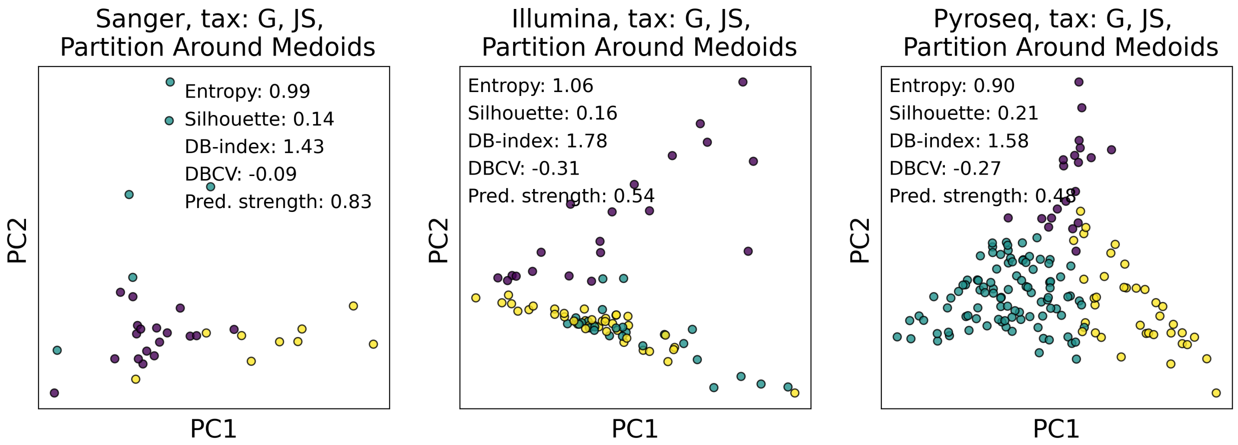

Figure S3: Visualization of the clustering partitions along with the metrics, for the Sanger, Illumina and Pyroseq datasets. The originally proposed clustering method was used - Partition Around Medoids (PAM) algorithm applied to the pre-computed pairwise Jensen-Shannon distances (JS).

the selected partitions, lying in the overlapping area, or by finding the closest one to the overlapping area. By taking into consideration all clustering metrics for every visualized partition, we conclude that there are no clustering partitions that meet all metrics criteria at the same time. The only partition demonstrated metrics that are closest to the optimal was obtained for the Sanger dataset using Silhouette score and the Davies-Bouldin index in Fig. S5 (top-left). This partition also reproduces the initially proposed enterotyping in [1]. Overall, according to the Adjusted Rand Index, the selected partitions found by our method for the Sanger and the Pyroseq datasets in Fig. S5, are moderately similar to the ones found by original approach from [1] and shown in Fig. S3. The clustering results for the Sanger, Illumina and Pyroseq datasets demonstrate lower Prediction Strength than for the clustering results on the large-scale AGP and HMP datasets. We emphasize that removing small percentage of the data should not change the structure of the data in an essential way, if there indeed exist natural clusters. Otherwise, unstable clusters can be attributed to artifacts of the data preprocessing or clustering method. The stability of partitions highly depends on the dataset size. If a dataset is sufficiently large, all variations of the structure are well-represented, reducing the influence of artifacts and outliers. Therefore, we hypothesize that the initial enterotypes findings [1] were possible due to the absence of available large metagenome datasets and the use of only linear methods of data analysis like PCA and the simple clustering methods like PAM.

Text S4 Pre-determined clustering

Here, we provide visualization and clustering metrics, using pre-determined artificial enterotype assignment for every data point. This assignment is based on the abundances of the *Bacteroides*, *Prevotella*, and *Ruminococcus* in different enterotypes from the Sanger dataset metagenomes [1]. Points whose OTU composition does not fall into any enterotype are not included in the visualization and clustering metrics calculation. We visualized the distribution of the data points in Fig. S6 (top) in three dimensions of the Genus taxonomy level, namely *Bacteroides*, *Prevotella*, and *Ruminococcus*, originally reported as the main enterotype drivers. We observe no clusters but a dense distribution of the data with high variance along these dimensions, which is also demonstrated by the PCA analysis in the manuscript. Application of such artificial partition to the three-dimensional projection in Fig. S6 (bottom) dissects the data distribution into three tightly arranged clusters that are driven mostly by the OTU coordinates. The corresponding clustering metrics indicate that such partition is stable and balanced, according to high values of the Prediciton Score and Entropy, yet poorly separated, according to the low DBCV index. We also demonstrate an apparent absence of natural clusters in such three-dimensional representations for the AGP and HMP dataseta by including them to the clustering step of our framework, that could potentially reveal possible partitions other than an artificial enterotype assignment. Further, we estimate clustering metrics for every representation of the data we obtain, given the artificial partition into enterotypes. These representations include manifold-learning embeddings, pairwise distances of the original data in different metric spaces, and projection on principal components, all in different taxonomy levels, namely Order, Family, and Genus. The results are presented in Fig. S7. As we see, such artificial assignment reveals no natural clusters in any data representation, according to the metrics thresholds.

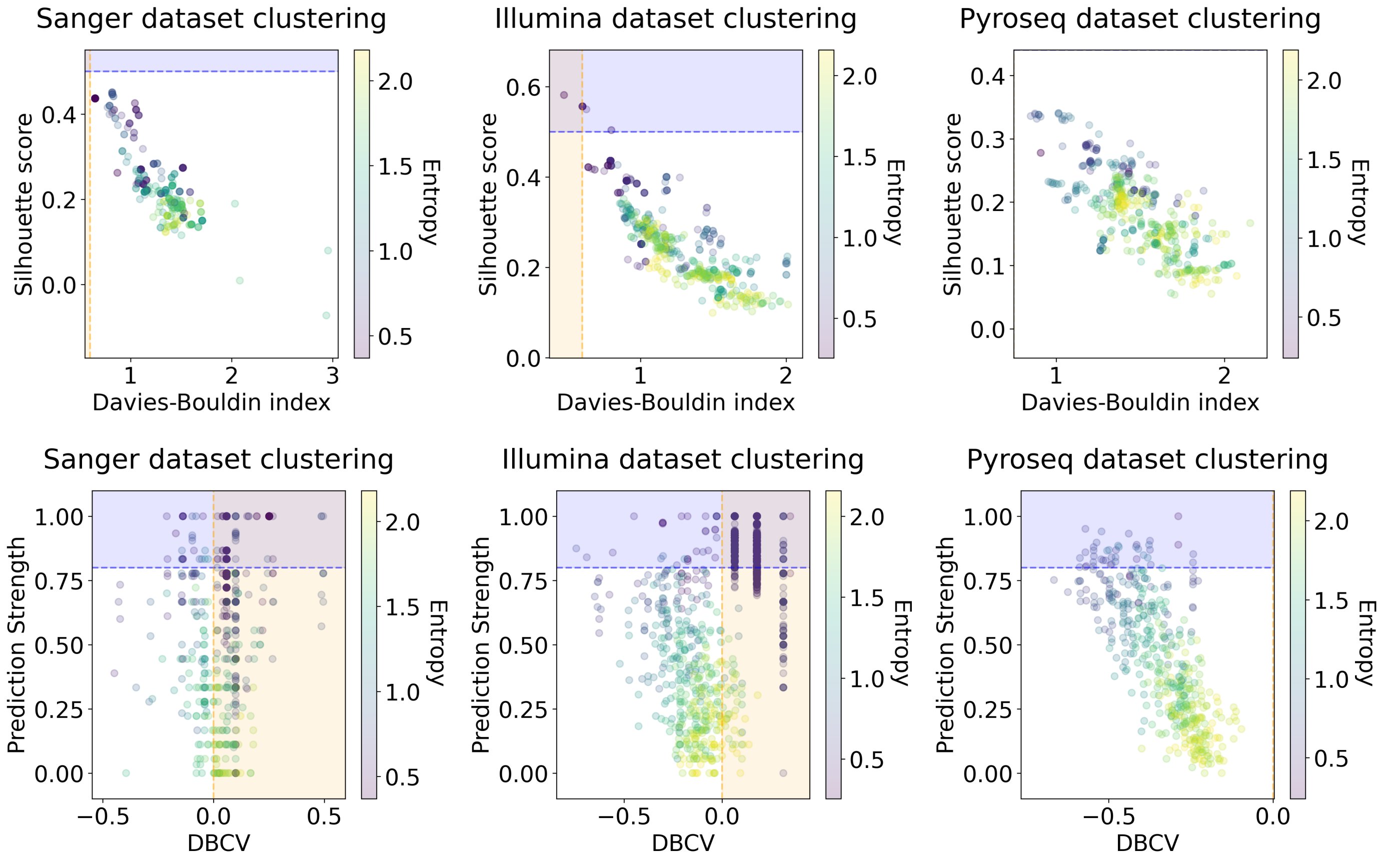

Figure S4: Silhouette score and Davies-Bouldin index (top), DBCV index and Prediction Strength (bottom) for all clustering partitions for Sanger, Illumina and Pyroseq datasets, obtained by our framework.

Text S5 Additional preprocessing

In this section we introduce results for the additional analysis, performed on the truncated AGP and HMP datasets, obtained after removing samples with an extreme gut microbiota composition containing more that 70% of the same OTU. This analysis includes all steps within our framework, namely, preprocessing, PCA projection, intrinsic dimension estimation, manifold learning and clustering, as described in Materials & Methods. The remained data percentages $n\%$ after samples removal are presented in Tab. S3. Dimensionalities of the truncated datasets at the Order, Family, and Genus taxonomy levels repeat the original ones (without removing OTUs accounting for $> 70\%$ abundance), as presented in Tab. 1 of the manuscript. Sorted cumulative sums of the singular values for d_{PCA} estimation are shown in Fig. S8 along with the PCA loadings, indicating contribution of the OTU coordinates to the principal components. Following the main analysis, we estimate the Median Absolute Error (MAE) and the Q_{loc} and Q_{glob} metrics of the linear projection on the principal components, that are presented in Tab. S3. As one can see in Fig. S8, the abundances of *Prevotella* and *Bacteroides* contribute most to the final principal components coordinates at the Genus taxonomy level. At the Family level, it is *Bacteroidaceae*, *Prevotellaceae*, and *Ruminococcaceae* for both datasets with *Enterobacteriaceae* and *Lactobacteriaceae* as additional strong drivers of the variance for HMP dataset. At the Order level it is *Bacteroidales* and *Clostridiales* for both datasets. After the PCA projection, we performed the intrinsic dimension estimation d_{MLE} and subsequent manifold learning procedure for the dimensionality reduction from d_{PCA} to d_{MLE}. Like in the original analysis, with all samples preserved, near-optimal manifold learning algorithms were found via enumeration of different combinations of potential hyperparameters and selecting the ones with the lowest reconstruction Median Absolute Error (MAE). The respective lowest MAE values of the data reconstruction from the nonlinear embedding are listed in Tab. S4 and the distribution of the Q_{loc} and Q_{glob} metrics is presented in Fig. S9.

In Fig. S10, we provide the distribution of metrics for all clustering results, calculated for every taxonomy level, sub-optimal manifold learning method, and clustering algorithm with different hyperparameters. Clustering partitions with moderate or strong support for both metrics correspond to the points lying at the intersection of the blue and orange areas. Consistent with the results from the main body of the manuscript, all found partitions consist of two or three highly imbalanced clusters with more than 95% of the data points concentrated in one cluster. We visualize

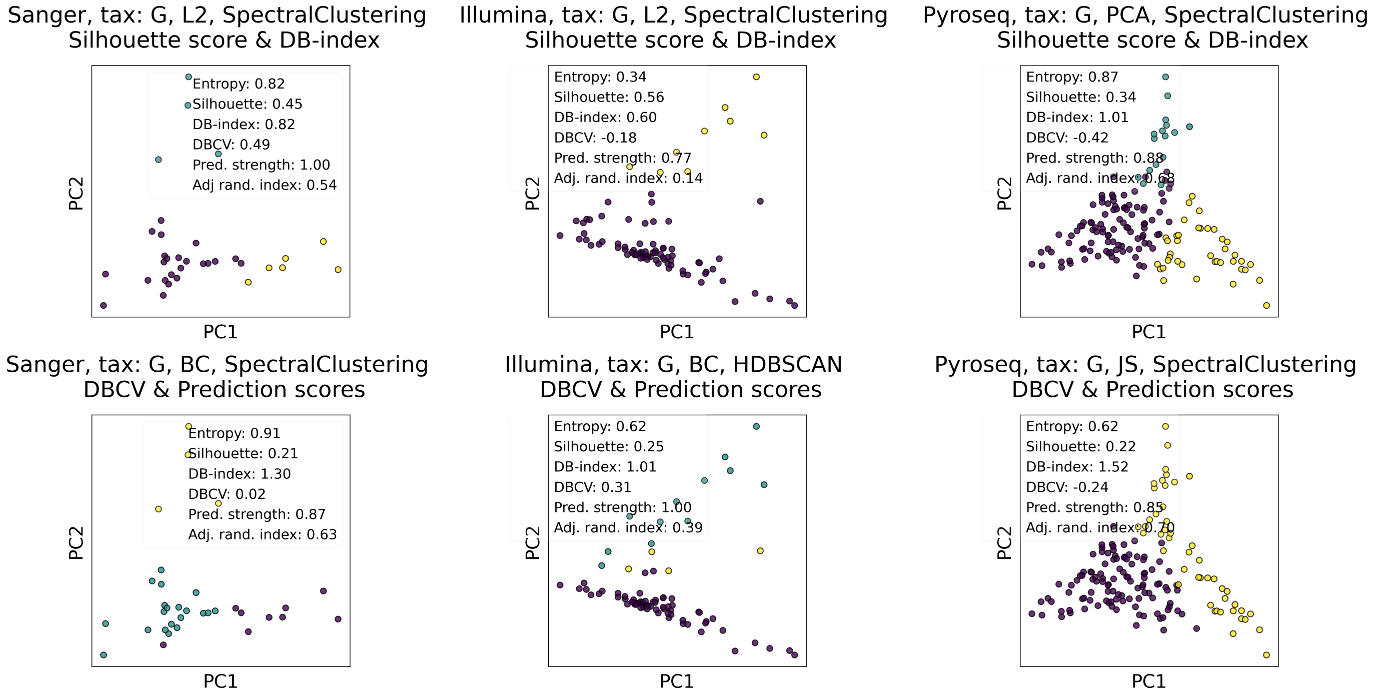

Figure S5: Visualization of the selected clustering partitions for the Sanger, Illumina and Pyroseq datasets in the two first principal components. Dataset name, taxonomy level, representation of the data, clustering algorithm, and the pair of metrics used to select the partition are shown in the title. Color indicates different clusters. Clustering metrics of the visualized partition are shown in the legend. Adjusted Rand Index represents similarity between the presented partition and the one obtained by the original method in Fig. S3.

Dataset	Tax	$n\%$	d_{PCA}	d_{MLE}	MAE	Q_{loc}	Q_{glob}
AGP	O	83	18	6	0.051	0.92	0.99
	F	96	34	8	0.040	0.96	0.99
	G	96	49	9	0.050	0.95	0.99
HMP	O	71	19	5	0.037	0.92	0.99
	F	79	39	7	0.037	0.94	0.99
	G	77	44	7	0.056	0.94	0.99

Table S3: Dimensionalities d_{PCA} - number of the first principal components explaining 99% variance, and d_{MLE} - estimated intrinsic dimension. $n\%$ - percentage of the data remained after removing samples with more that 70% of composition occupied by the same OTU. MAE - Median Absolute Error of the linear inverse transformation from data projected on the principal components to the original space of taxon abundances. Q_{loc} and Q_{glob} - metrics that indicate preservation of the local and global data structure correspondingly.

selected partitions with moderate support in Fig. S11, by projecting on first two principal components or by using Large Margin Nearest Neighbor method [13]. For visualization, for every pair of metrics and the dataset, we select the partition, with the maximal entropy, among all partitions satisfying metrics thresholds. As one can see, the clusters are non-convex, yet distinguishable, which is consistent with the metrics. Nevertheless, the distribution of the data points among the clusters for every selected partition is highly imbalanced, which is indicated by the low entropy value. As in the main analysis, these results imply that diverse clustering methods applied to different manifold learning algorithms yield stable and distinctive, but highly imbalanced partitions. We assign such partitions to the data outliers or artifacts of the manifold learning algorithms and clustering methods, since clusters that contain less than 5% of the total data are not related to the enterotypes. In Fig. S12 and S13, we demonstrate continuous variation of the specific OTU at the Genus taxonomy level, along a two-dimensional representation, obtained by the UMAP and t-SNE methods. The points are colored as specific taxon relative abundances, corresponding to the genera of bacteria most relevant for the definition of enterotypes, *Bacteroides*, *Prevotella*, and *Ruminococcus*, according to the initial finding [1]. Salient parts of the manifolds represent higher concentrations of the *Bacteroides* and *Prevotella* OTUs. Prior to the UMAP and t-SNE dimensionality reduction, datasets were projected on the principal components capturing 99% of variance. After obtaining two-dimensional representations, small clusters of points containing less that 1% of the data were removed

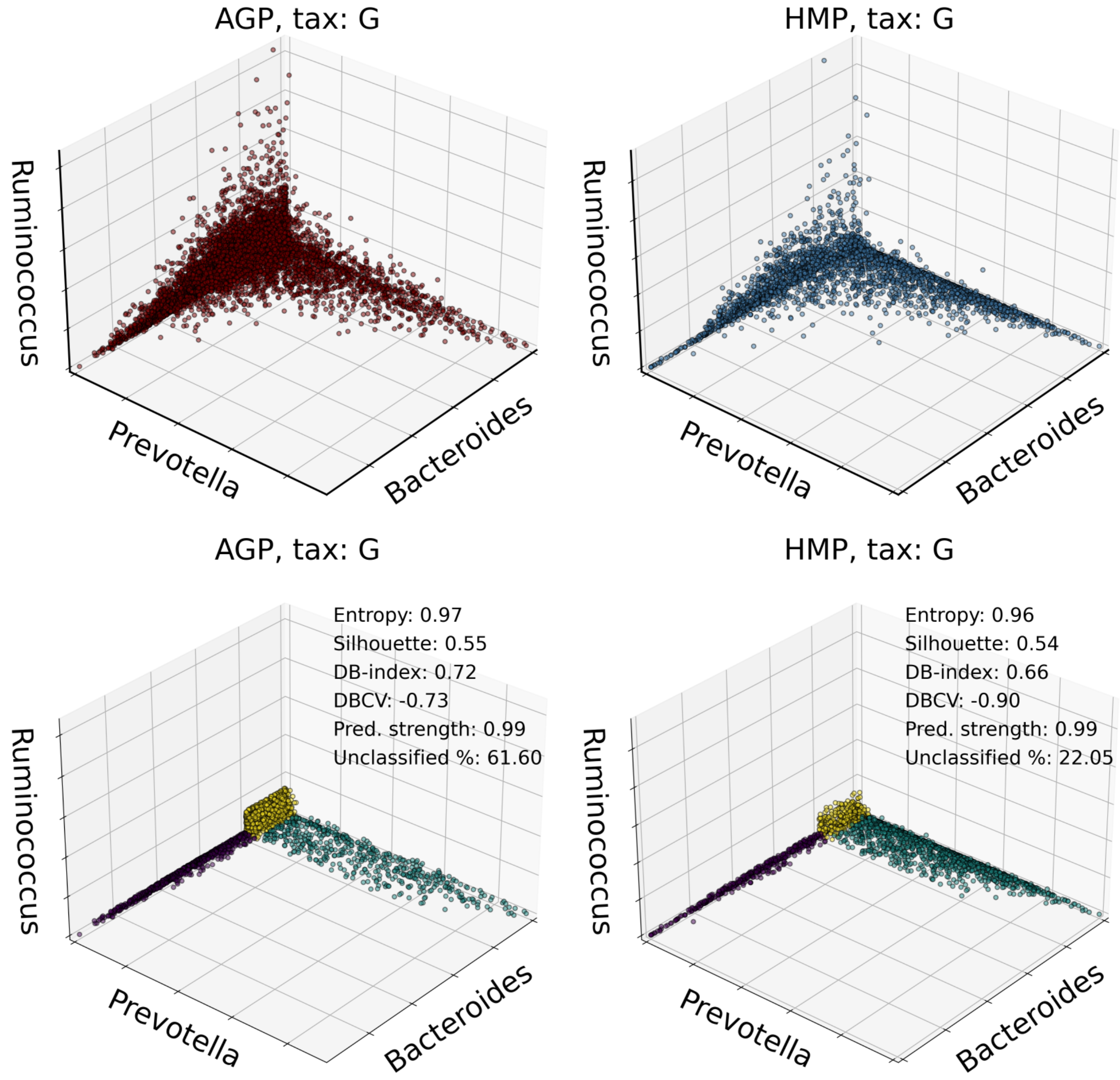

Figure S6: Visualization of the AGP and HMP datasets in projection on three main enterotype drivers, namely *Bacteroides*, *Prevotella* and *Ruminococcus* (top). The same visualization, but with an artificial clustering partition applied (bottom). This partition is provided by assigning each point to corresponding enterotype. Points that do not fall into any enterotype are denoted as Unclassified and are not included in the visualization. Corresponding clustering metrics, estimated for an artificial partition are presented in the legend.

using the Local Outlier Factor algorithm [2]. Further, for the two-dimensional visualizations, we estimate the density of the points. We perform standard Kernel Density Estimation (KDE) with the bandwidth parameter equal to the median value of a distribution of pairwise distances from every point to 100 closest neighbors. As we see in Fig. S14, the density of regions is not uniform, indicating that there are regions of preferential data concentration in UMAP and t-SNE visualizations in Fig. S12 and Fig. S13, correspondingly. Removing all regions in Fig. S14 with density less than the 70% percentile of the total density distribution, we obtain well-separated, high-density regions. In Fig. S15, we show the arrangement of those regions in the two-dimensional visualization (Z_1 and Z_2 coordinates), along with the distributions of ten most significant OTU in each region. The most significant OTUs are the ones with the highest mean value for all selected high-density regions. We observe that the difference in the OTU distribution between the high-density clusters is mainly controlled by variation of *Bacteroides*, *Prevotella*, *Ruminococcus*, *Lactobacillus*, and *Faecalibacterium*, as well as variation of an unclassified OTU in the Genus level, denoted as *Rest*. This observation is consistent with the OTU abundance gradient visualization in Fig. S13 and Fig. S12 and the similar analysis performed for the data with all samples retained in the main body of the manuscript.

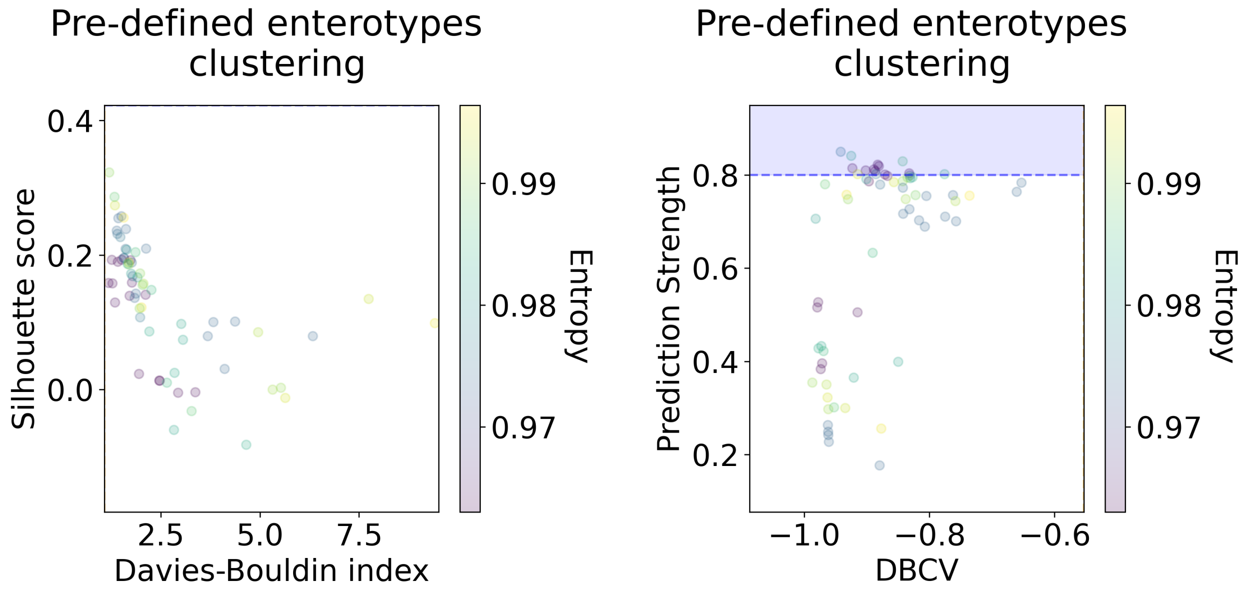

Figure S7: Distribution of the Silhouette score and Davies-Bouldin index (left), DBCV index and Prediction Strength (right) of an artificial enterotyping partition applied to the different representations of the AGP and HMP datasets: pre-computed distances, low-dimensional manifold learning embeddings and projection on principal components.

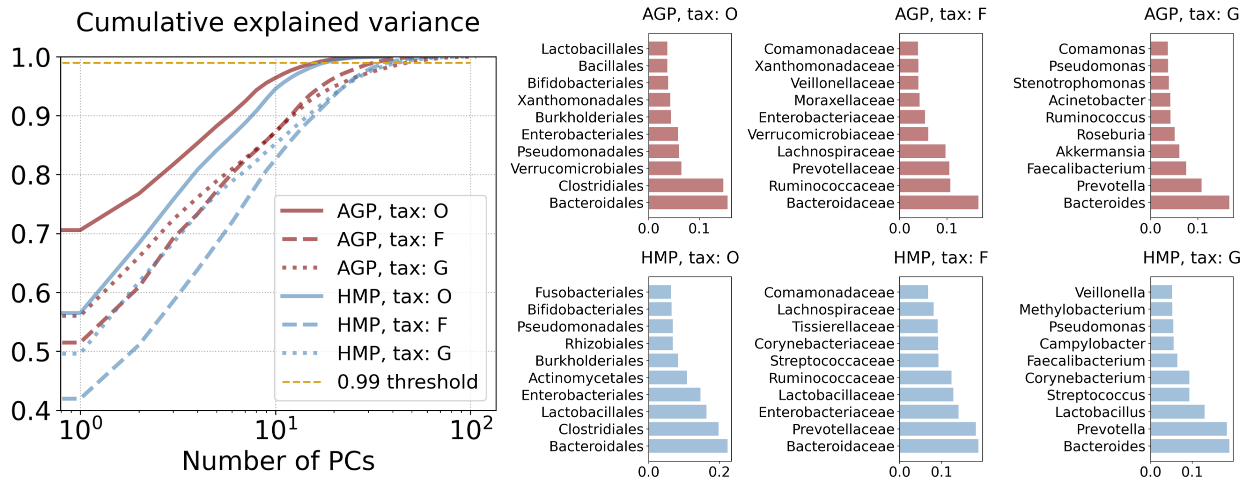

Figure S8: Cumulative explained variance of the Principal Component Analysis (PCA) and PCA loadings, representing contribution of original taxonomy coordinates to the principal components, for AGP and HMP datasets in different taxonomy levels: O - Order, F - Family, G - Genus.

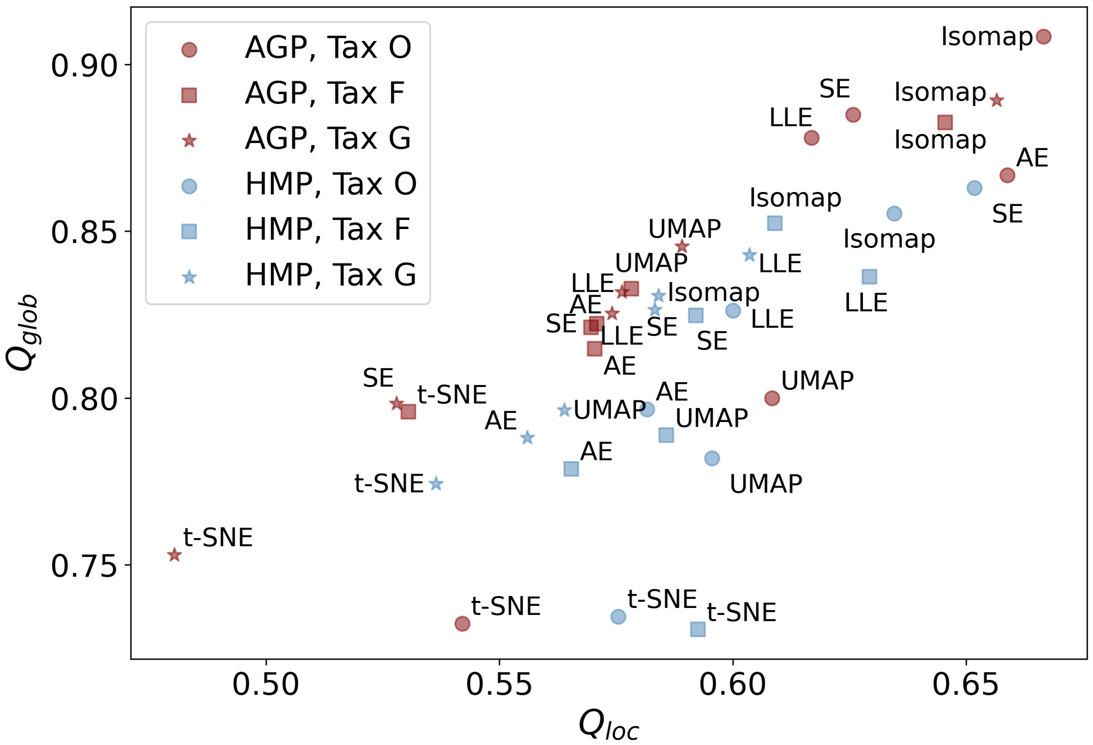

Figure S9: Co-ranking metrics for different manifold learning methods. Horizontal axis - Q_{loc} metric (local information preservation), vertical axis - Q_{glob} metric (global information preservation) of the non-linear dimensionality reduction methods. Datasets (AGP and HMP) and taxonomy levels (O - Order, F - Family, G - Genus) are shown in the inset. SE - Spectral Embedding, LLE - Locally Linear Embedding, AE - AutoEncoder.

Dataset	Method	Tax O	Tax F	Tax G
AGP	AutoEncoder	0.06	0.19	0.22
	t-SNE	0.05	0.19	0.22
	UMAP	0.06	0.22	0.24
	Isomap	0.06	0.22	0.25
	LLE	0.06	0.21	0.24
	Spectral	0.06	0.21	0.23
HMP	AutoEncoder	0.09	0.24	0.22
	t-SNE	0.13	0.28	0.27
	UMAP	0.14	0.31	0.30
	Isomap	0.15	0.34	0.33
	LLE	0.15	0.32	0.31
	Spectral	0.17	0.37	0.34

Table S4: The Median Absolute Error of the reconstruction, assessed using Leave-One-Out procedure. The reconstruction is done by the independent K-Nearest Neighbors Regression of the coordinates in the original space of relative taxon abundances from the non-linear embedding.

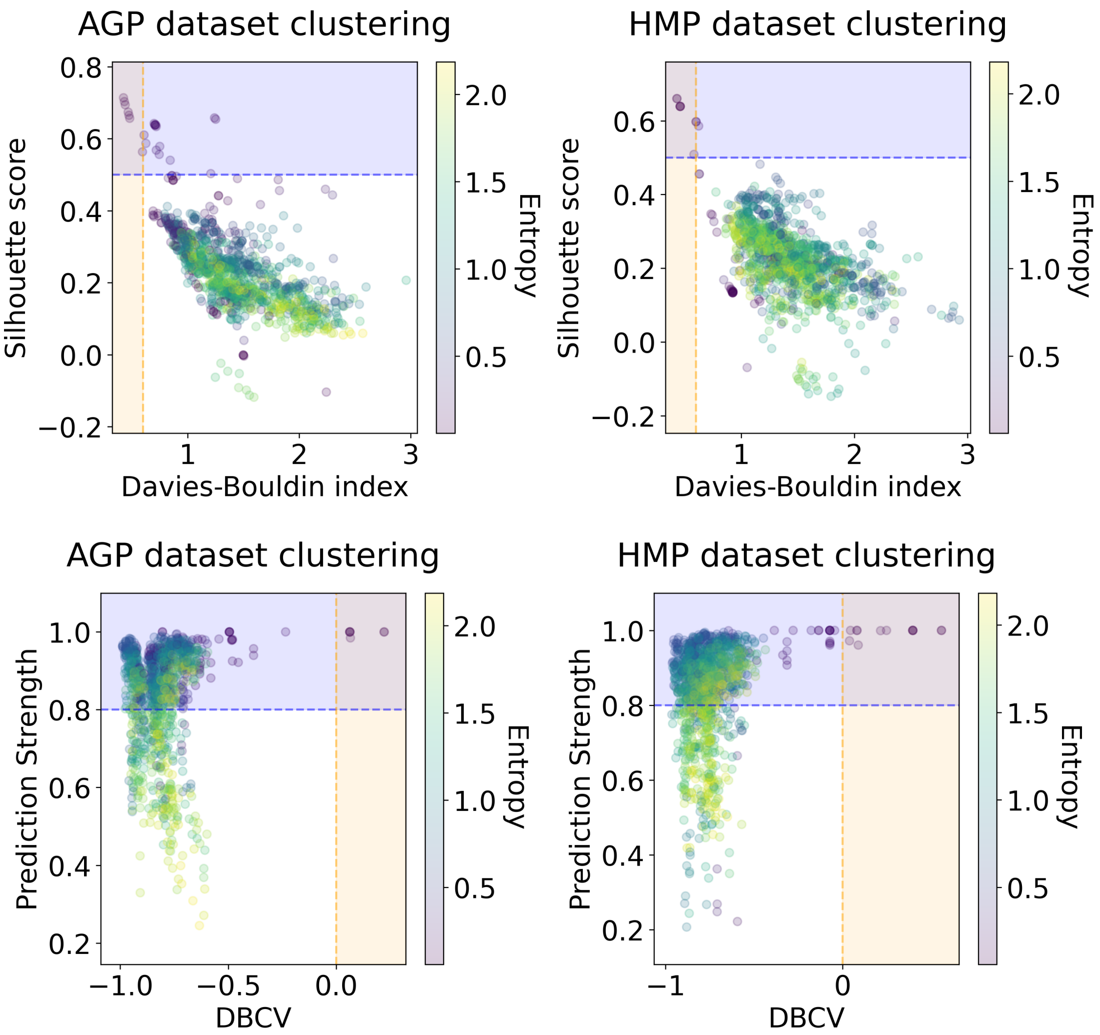

Figure S10: Silhouette score and Davies-Bouldin index (top), DBCV index and Prediction Strength (bottom) of clustering partitions for the truncated AGP and HMP datasets (the samples with the same OTU constituting more than 70% of the microbiome composition removed).

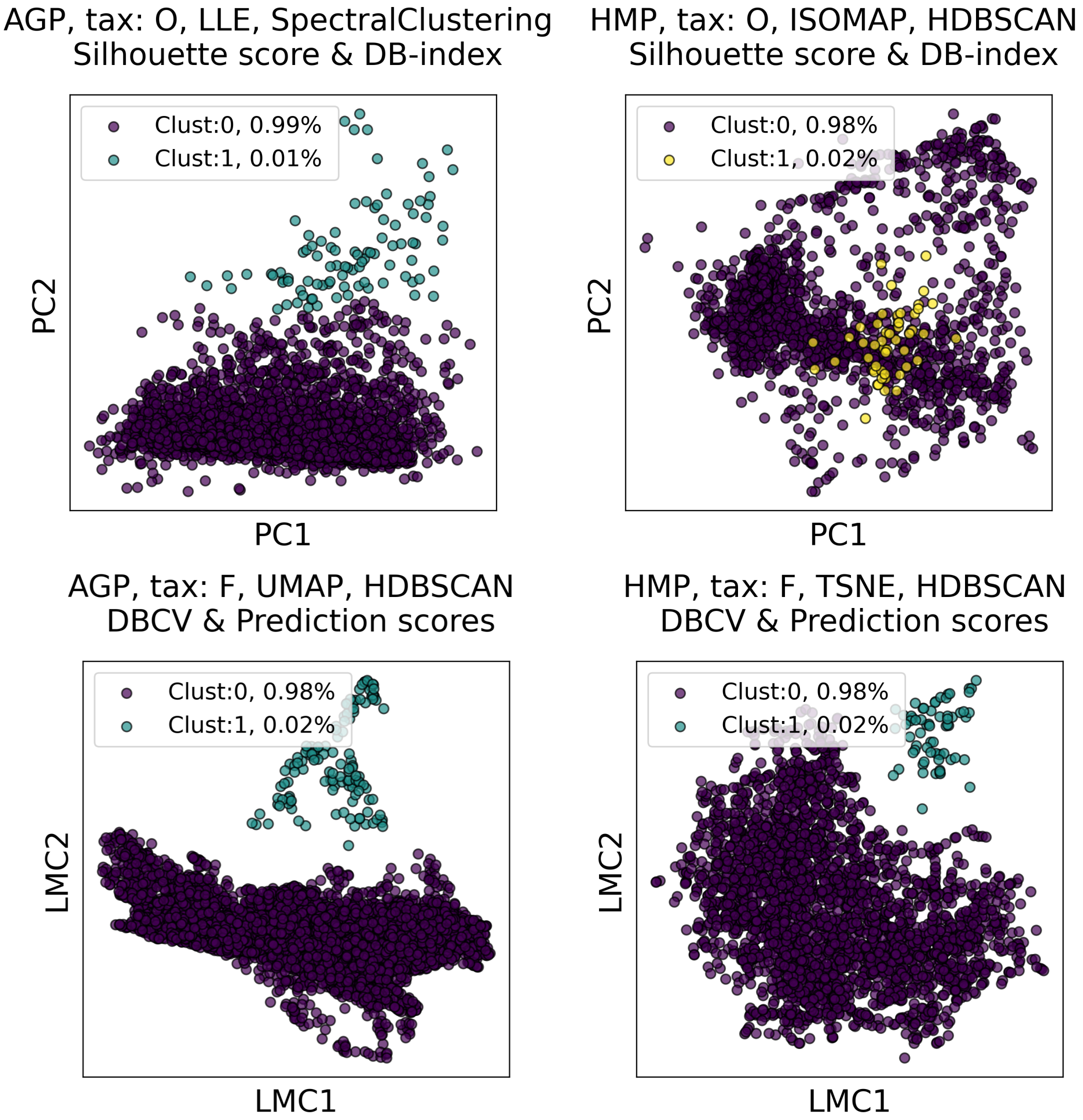

Figure S11: Visualization of the clustering results for the AGP and HMP datasets, using the first two principal components (PC1 and PC2) or the Large Margin Nearest Neighbor method (LMC1 and LMC2). The visualized clustering partitions have the highest entropy among all partitions satisfying corresponding metrics thresholds. Dataset name, taxonomy level, representation of the data, clustering algorithm, and the pair of metrics used to select the partition are shown in the title. Color indicates different clusters. Percentage of the data belonging to each cluster is depicted on the legend.

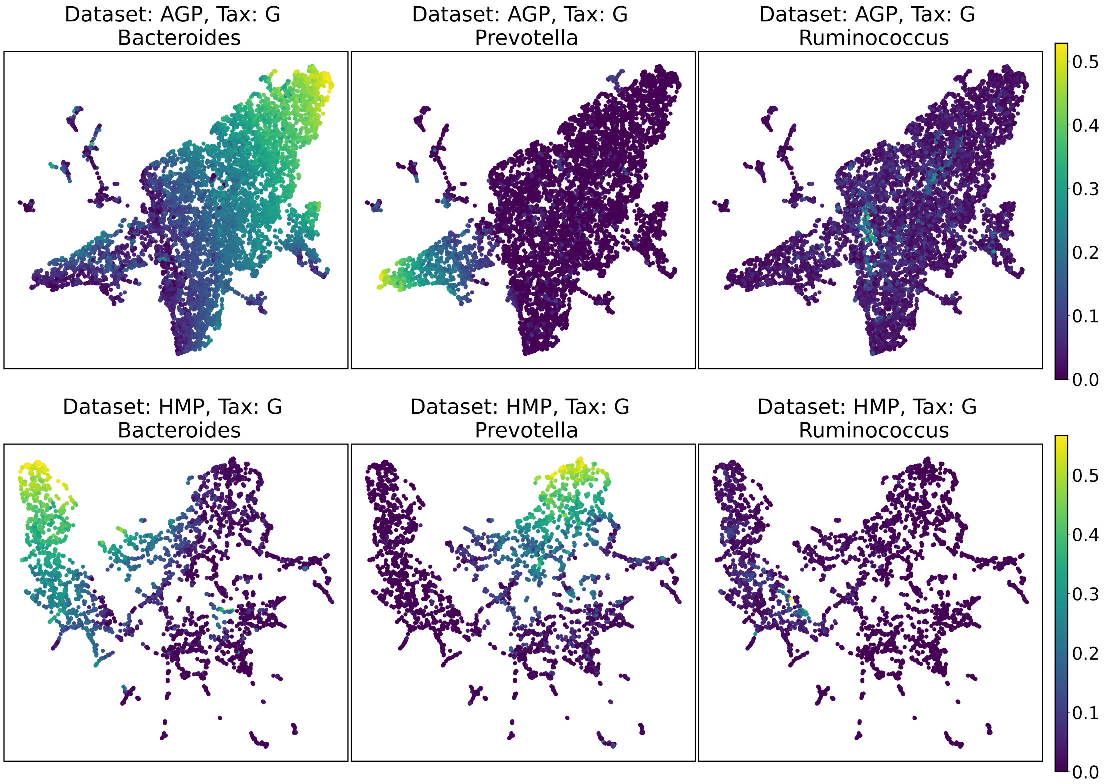

Figure S12: Two-dimensional UMAP visualization of truncated AGP and HMP datasets for the Genus taxonomy level. Samples with extreme gut microbiota composition that includes more that 70% of the same OTU were removed prior to the visualization. Colors reflect the relative abundance of specific taxa, see the headers.

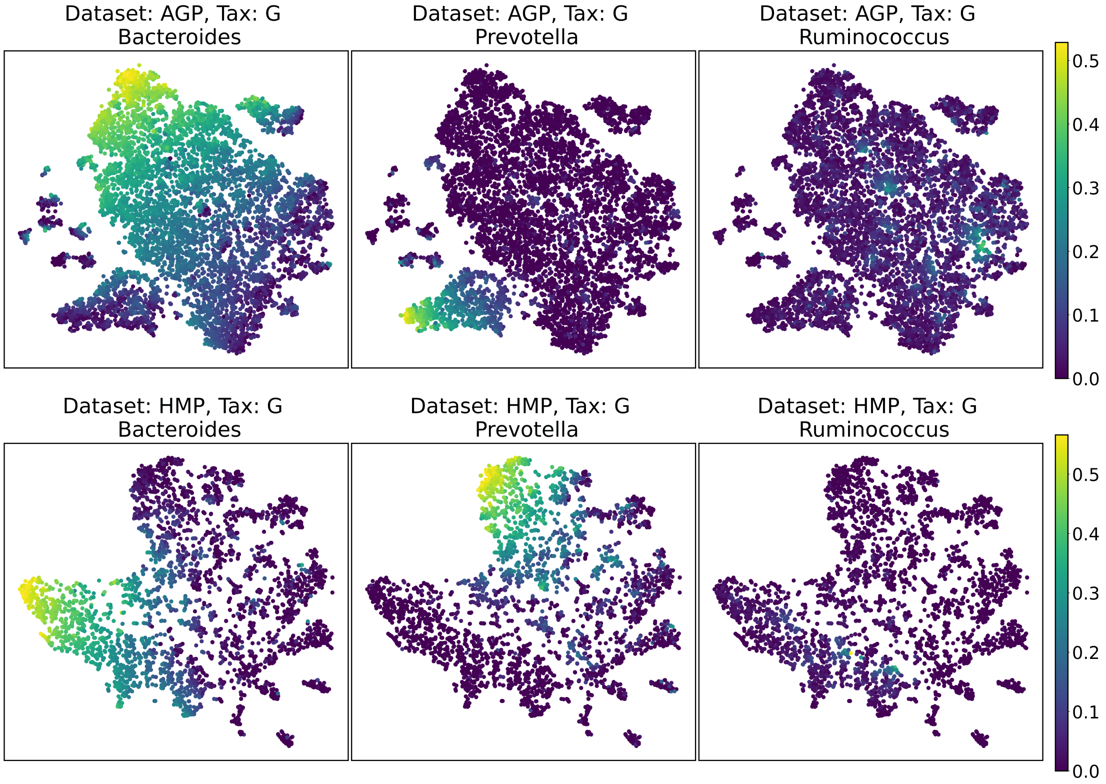

Figure S13: Two-dimensional t-SNE visualization of truncated AGP and HMP datasets for the Genus taxonomy level. Samples with extreme gut microbiota composition that includes more that 70% of the same OTU were removed prior to the visualization. Colors reflect the relative abundance of specific taxa, see the headers.

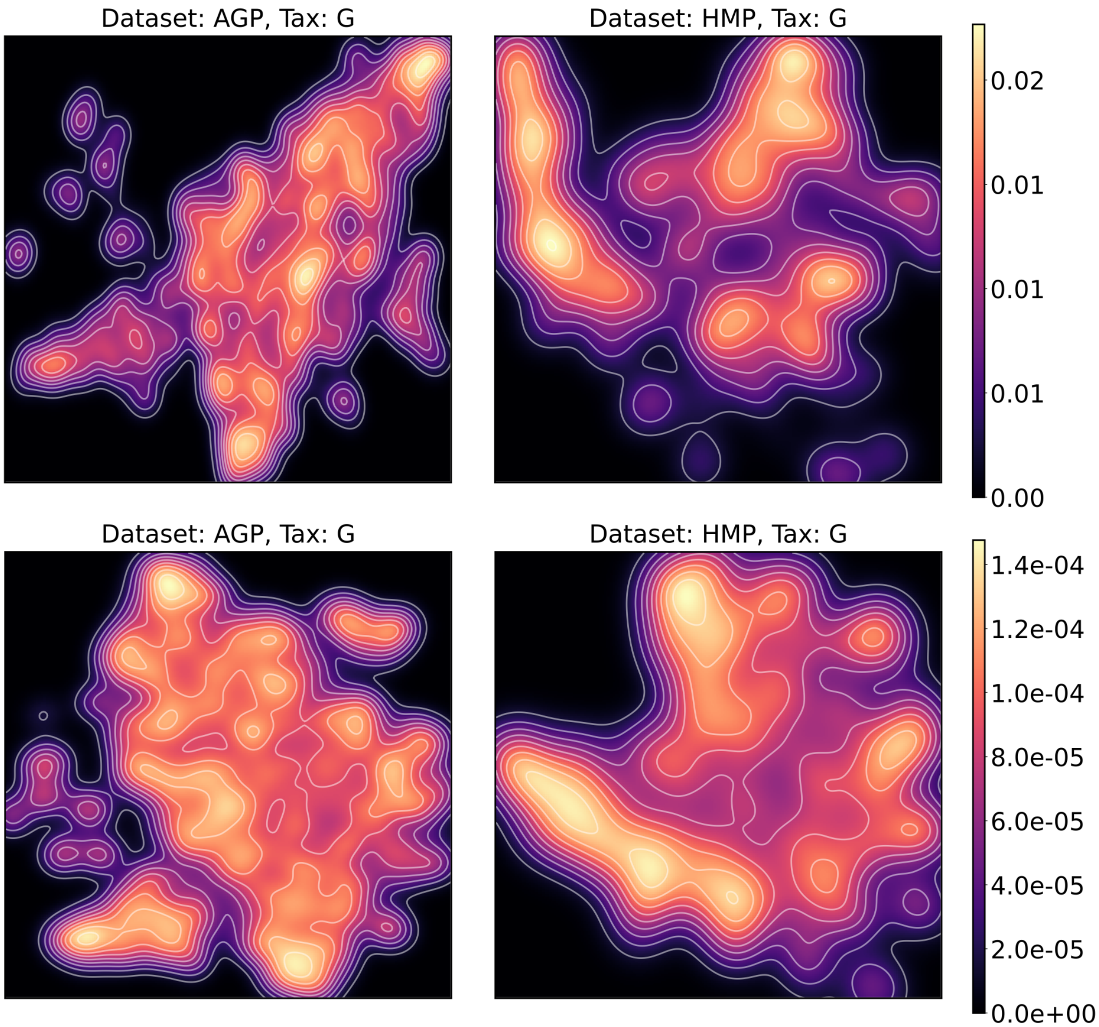

Figure S14: Kernel Density Estimation of UMAP (top) and t-SNE (bottom) two-dimensional visualizations. Density color indicates relative likelihood of the point to belong to the data distribution, according to KDE.

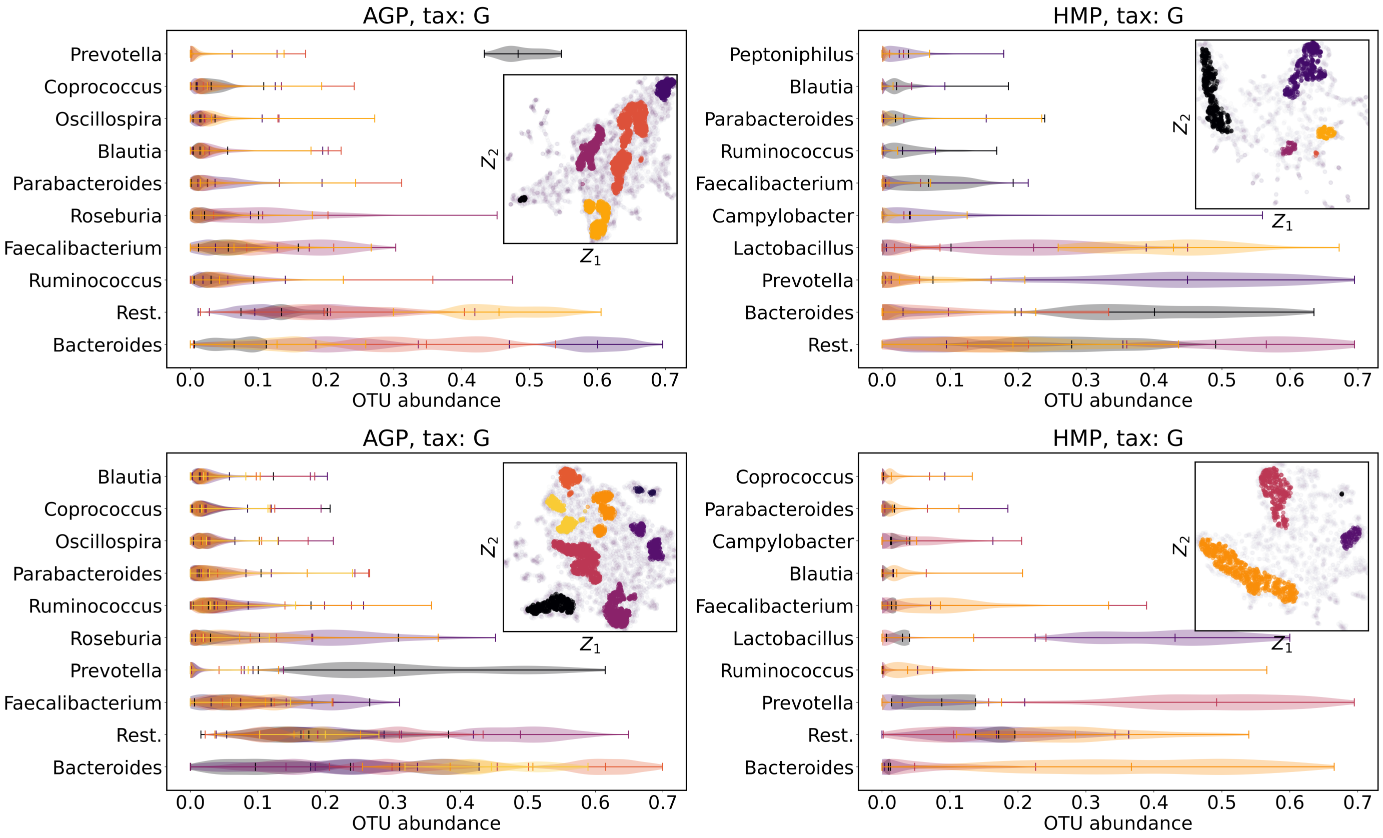

Figure S15: Analysis of the high-density regions for UMAP (top) and t-SNE (bottom) two-dimensional visualizations. The regions correspond to the Kernel Density Estimation likelihood larger than 70% percentile of the total likelihood distribution. Color indicates different high-density clusters, depicted in the two-dimensional scatter plot with Z_1 and Z_2 coordinates. For the first ten selected OTU with the largest mean value among all high-density regions, the violin plots depict their distribution within each region.

Algorithm	Description	Pros	Cons
Spectral Embedding	Uses eigenvalues and eigenvectors of the Laplacian matrix based on dataset graph to represent dataset while preserving its spectral properties	Captures nonlinear data structure, preserves local properties, computationally efficient, explicitly emphasizes clusters	Requires hyperparameters tuning, may not work well for large datasets and accentuate spurious clusters, sensitive to noise
Locally Linear Embedding (LLE)	Locally preserves linear structure of the data by reproducing it in the lower-dimensional space	Captures nonlinear data structure, computationally efficient, requires no assumptions about data	Preserve only local properties, may not work well for high-dimensional data, sensitive to the choice of the hyperparameters
ISOMAP	Calculates geodesic distances in a neighborhood graph to preserve the intrinsic geometry of the data in the embedding	Captures nonlinear data structure, works well for data with manifold structures, robust to noise	Requires tuning of hyperparameters, may not work well for data with disconnected point clouds
Autoencoder	Neural network that learns to encode and decode data, effectively reducing dimensionality	Captures nonlinear data structure, learns inverse transform from embedding space to the original	Requires training data and may suffer from overfitting, sensitive to the noise and outliers
t-SNE	Uses a probabilistic approach and optimization to create an embedding preserving pairwise similarities between points	Captures local and global nonlinear structure, works well for the high-dimensional data with intricate structure	Computationally expensive, sensitive to the choice of hyperparameters, embeddings are not always interpretable
UMAP	Uses a fuzzy topological representation of the data to create a low-dimensional embedding, using optimization procedure similar to t-SNE	Captures nonlinear data structure, works well for high-dimensional data with a complex structure, less computationally expensive than t-SNE	Relies on assumptions about the data, embeddings are not always interpretable and depend on the hyperparameters

Table S5: Comparison of the dimensionality reduction algorithms used in the manuscript.

Algorithm	Description	Pros	Cons
Partition Around Medoids (PAM)	Finds a set of medoids, one for every cluster. Every medoid minimizes dissimilarity between itself and the points within the corresponding cluster	Fast, robust to outliers, handles categorical data, provides interpretable cluster representatives, straightforwardly addresses out of sample data	Requires specifying number of clusters, sensitive to the initialization, generally does not account for the non-convex, density-based clusters, and noise
Spectral Clustering	Treats clustering as a graph partition problem, by using the eigenvalues and eigenvectors of the the Laplacian matrix based on dataset graph	Capable of revealing non-convex and noisy clusters with different sizes and shapes, efficient for sparse affinity matrices	Requires specifying number of clusters and tuning of hyperparameters, sensitive to outliers, does not account for noise, computationally expensive
HDBSCAN	Identifies clusters by estimating density of the data, constructing hierarchy of clusters and condensing small clusters	Robust to noise, varying shapes, densities, and sizes of the clusters, works well for datasets with non-convex clusters	Highly sensitive to the choice of hyperparameters that depend on the data, computationally expensive for large datasets

Table S6: Comparison of the clustering algorithms used in the manuscript.

References

[1] Manimozhiyan Arumugam, Jeroen Raes, Eric Pelletier, Denis Le Paslier, Takuji Yamada, Daniel R. Mende, Gabriel R. Fernandes, Julien Tap, Thomas Bruls, Jean-Michel Batto, Marcelo Bertalan, Natalia Borruel, Francesc Casellas, Leyden Fernandez, Laurent Gautier, Torben Hansen, Masahira Hattori, Tetsuya Hayashi, Michiel Kleerebezem, Ken Kurokawa, Marion Leclerc, Florence Levenez, Chaysavanh Manichanh, H. Bjørn Nielsen, Trine Nielsen, Nicolas Pons, Julie Poulain, Junjie Qin, Thomas Sicheritz-Ponten, Sebastian Tims, David Torrents, Edgardo Ugarte, Erwin G. Zoetendal, Jun Wang, Francisco Guarner, Oluf Pedersen, Willem M. de Vos, Søren Brunak, Joel Doré, Jean Weissenbach, S. Dusko Ehrlich, and Peer Bork. Enterotypes of the human gut microbiome. *Nature*, 473(7346):174–180, may 2011.

[2] Markus M. Breunig, Hans-Peter Kriegel, Raymond T. Ng, and Jörg Sander. Lof: Identifying density-based local outliers. In *Proceedings of the 2000 ACM SIGMOD International Conference on Management of Data*, SIGMOD '00, page 93–104, New York, NY, USA, 2000. Association for Computing Machinery.

[3] David L. Davies and Donald W. Bouldin. A Cluster Separation Measure. *IEEE Transactions on Pattern Analysis and Machine Intelligence*, PAMI-1(2):224–227, 1979.

[4] John A. Hartigan, Helmut Spath, and J. Van Ryzin. Clustering Algorithms, nov 1981.

[5] Lawrence Hubert and Phipps Arabie. Comparing partitions. *Journal of Classification*, 2(1):193–218, dec 1985.

[6] Omry Koren, Dan Knights, Antonio Gonzalez, Levi Waldron, Nicola Segata, Rob Knight, Curtis Huttenhower, and Ruth E. Ley. A Guide to Enterotypes across the Human Body: Meta-Analysis of Microbial Community Structures in Human Microbiome Datasets. *PLoS Computational Biology*, 9(1):e1002863, jan 2013.

[7] John A. Lee and Michel Verleysen. Scale-independent quality criteria for dimensionality reduction. *Pattern Recognition Letters*, 31(14):2248–2257, oct 2010.

[8] Davoud Moulavi, Pablo A. Jaskowiak, Ricardo J. G. B. Campello, Arthur Zimek, and Jörg Sander. Density-Based Clustering Validation. In *Proceedings of the 2014 SIAM International Conference on Data Mining*, volume 2 of *Proceedings*, pages 839–847. Society for Industrial and Applied Mathematics, Philadelphia, PA, apr 2014.

[9] William M. Rand. Objective criteria for the evaluation of clustering methods. *Journal of the American Statistical Association*, 66(336):846–850, 1971.

[10] Peter J. Rousseeuw. Silhouettes: A graphical aid to the interpretation and validation of cluster analysis. *Journal of Computational and Applied Mathematics*, 20(C):53–65, nov 1987.

[11] C. E. Shannon. A Mathematical Theory of Communication. *Bell System Technical Journal*, 27(3):379–423, jul 1948.

[12] Robert Tibshirani and Guenther Walther. Cluster Validation by Prediction Strength. *Journal of Computational and Graphical Statistics*, 14(3):511–528, sep 2005.

[13] Kilian Q Weinberger, John Blitzer, and Lawrence Saul. Distance metric learning for large margin nearest neighbor classification. In Y. Weiss, B. Schölkopf, and J. Platt, editors, *Advances in Neural Information Processing Systems*, volume 18. MIT Press, 2005.
